# Supplementary material for: Hospital Admission Patterns in Adult Patients with Community-Acquired Pneumonia Who Received Ceftriaxone and a Macrolide by Disease Severity across United States Hospitals
Source: Antibiotics (Basel). 2020 Sep 4;9(9):577. doi: 10.3390/antibiotics9090577 (PMC7557926; doi:10.3390/antibiotics9090577)
Supplement: Supplementary file 1 [file antibiotics-09-00577-s001.pdf]

Supplementary Data

Table S1. ICD-9-CM codes for CAP.

ICD-9-CM	Code Description
481	Pneumococcal pneumonia [*Streptococcus pneumoniae* pneumonia]
4821	Pneumonia due to *Pseudomonas*
4822	Pneumonia due to *Haemophilus influenzae*
48230	Pneumonia due to *Streptococcus*, unspecified
48231	Pneumonia due to *Streptococcus*, group A
48232	Pneumonia due to *Streptococcus*, group B
48239	Pneumonia due to other *Streptococcus*
48240	Pneumonia due to *Staphylococcus*, unspecified
48241	Methicillin susceptible pneumonia due to *Staphylococcus aureus*
48242	Methicillin resistant pneumonia due to *Staphylococcus aureus*
48249	Other *Staphylococcus* pneumonia
48281	Pneumonia due to anaerobes
48282	Pneumonia due *to Escherichia coli*
48283	Pneumonia due to other gram-negative bacteria
48284	Pneumonia due to Legionnaires' disease
48289	Pneumonia due to other specified bacteria
4829	Bacterial pneumonia, unspecified
4830	Pneumonia due to *Mycoplasma pneumoniae*
4831	Pneumonia due to *Chlamydia*
4838	Pneumonia due to other specified organism
4845	Pneumonia in anthrax
485	Bronchopneumonia, organism unspecified
486	Pneumonia, organism unspecified

Abbreviation: ICD-9-CM, International Classification of Diseases, 9th revision, Clinical Modification.

Table S2. MedAssets PSI score criteria and adaptations.

PSI Score Criteria	Formula to Calculate Score (present = 1; absent = 0)	Corresponding Conditions with Available ICD-9-CM Code
Age	Age at index in years +	
Gender	Female *(−10) +	
Nursing home resident	Nursing home resident *10 +	Excluded from the analysis
Neoplastic disease	Neoplastic disease *30 +	Neoplastic disease
Liver disease history	Liver disease history *20 +	Liver disease before or at index date
CHF history	CHF history *10 +	CHF before or at index date
Cerebrovascular disease history	Cerebrovascular disease history *10 +	Cerebrovascular disease before or at index date
Renal disease history	Renal disease history *10 +	Renal disease before or at index date
Altered mental status	Altered mental status *20 +	Altered mental status
Respiratory rate >29 breaths/minute	[Respiratory rate >29 breaths/minute] *20 +	Tachypnea
SBP <90 mm Hg	[SBP <90 mm Hg] *20 +	Hypotension
Temperature <35°C (95°F) or >39.9°C (103.8°F)	[Temperature <35°C (95°F) or >39.9°C (103.8°F)] *15 +	Hypothermia, malignant hyperthermia
Pulse >124 beats/minute	[Pulse >124] *10 +	Tachycardia
pH <7.35	[pH <7.35] *30 +	Acidosis
BUN >29 mg/dL	[BUN >29] *20 +	Non-specific abnormal results of function study of kidney
Sodium < 130 mg/dL	[Sodium <130] *20 +	Hypoosmolality and/or hyponatremia
Glucose > 249 mg/dL (US) or 13.8 mmol/L (SI)	[Glucose >249 (US) or 13.8 (SI)] *10 +	Abnormal glucose
Hct 30%	[Hct <30%] *10 +	Precipitous drop in Hct
Partial pressure of oxygen <60 mm Hg	[Partial pressure of oxygen <60 mm Hg] *10 +	Hypoxemia
Pleural effusion on X-ray	[Pleural effusion on X-ray] *10 +	Pleural effusion

BUN, blood urea nitrogen; CHF, congestive heart failure; Hct, hematocrit; PSI, Pneumonia Severity Index; ICD-9-CM, International Classification of Diseases, 9th revision, clinical modification (ICD-9-CM); SBP, systolic blood pressure.

Table S3. ICD-9-CM codes for PSI score criteria and adaptations.

PSI Score Criteria	ICD-9-CM	Description
Neoplastic disease	140	Malignant neoplasm of lip
Neoplastic disease	1400	Malignant neoplasm of upper lip vermilion border
Neoplastic disease	1401	Malignant neoplasm of lower lip vermilion border
Neoplastic disease	1403	Malignant neoplasm of upper lip inner aspect
Neoplastic disease	1404	Malignant neoplasm of lower lip inner aspect
Neoplastic disease	1405	Malignant neoplasm of lip unspecified inner aspect
Neoplastic disease	1406	Malignant neoplasm of commissure of lip
Neoplastic disease	1408	Malignant neoplasm of other sites of lip
Neoplastic disease	1409	Malignant neoplasm of lip unspecified vermilion border
Neoplastic disease	141	Malignant neoplasm of tongue
Neoplastic disease	1410	Malignant neoplasm of base of tongue
Neoplastic disease	1411	Malignant neoplasm of dorsal surface of tongue
Neoplastic disease	1412	Malignant neoplasm of tip and lateral border of tongue
Neoplastic disease	1413	Malignant neoplasm of ventral surface of tongue
Neoplastic disease	1414	Malignant neoplasm of anterior two-thirds of tongue part unspecified
Neoplastic disease	1415	Malignant neoplasm of junctional zone of tongue
Neoplastic disease	1416	Malignant neoplasm of lingual tonsil
Neoplastic disease	1418	Malignant neoplasm of other sites of tongue
Neoplastic disease	1419	Malignant neoplasm of tongue unspecified
Neoplastic disease	142	Malignant neoplasm of major salivary glands
Neoplastic disease	1420	Malignant neoplasm of parotid gland
Neoplastic disease	1421	Malignant neoplasm of submandibular gland
Neoplastic disease	1422	Malignant neoplasm of sublingual gland
Neoplastic disease	1428	Malignant neoplasm of other major salivary glands
Neoplastic disease	1429	Malignant neoplasm of salivary gland unspecified
Neoplastic disease	143	Malignant neoplasm of gum
Neoplastic disease	1430	Malignant neoplasm of upper gum
Neoplastic disease	1431	Malignant neoplasm of lower gum
Neoplastic disease	1438	Malignant neoplasm of other sites of gum
Neoplastic disease	1439	Malignant neoplasm of gum unspecified
Neoplastic disease	144	Malignant neoplasm of floor of mouth
Neoplastic disease	1440	Malignant neoplasm of anterior portion of floor of mouth
Neoplastic disease	1441	Malignant neoplasm of lateral portion of floor of mouth
Neoplastic disease	1448	Malignant neoplasm of other sites of floor of mouth
Neoplastic disease	1449	Malignant neoplasm of floor of mouth part unspecified
Neoplastic disease	145	Malignant neoplasm of other and unspecified parts of mouth
Neoplastic disease	1450	Malignant neoplasm of cheek mucosa
Neoplastic disease	1451	Malignant neoplasm of vestibule of mouth
Neoplastic disease	1452	Malignant neoplasm of hard palate
Neoplastic disease	1453	Malignant neoplasm of soft palate
Neoplastic disease	1454	Malignant neoplasm of uvula
Neoplastic disease	1455	Malignant neoplasm of palate unspecified
Neoplastic disease	1456	Malignant neoplasm of retromolar area
Neoplastic disease	1458	Malignant neoplasm of other specified parts of mouth
Neoplastic disease	1459	Malignant neoplasm of mouth unspecified
Neoplastic disease	146	Malignant neoplasm of oropharynx
Neoplastic disease	1460	Malignant neoplasm of tonsil
Neoplastic disease	1461	Malignant neoplasm of tonsillar fossa
Neoplastic disease	1462	Malignant neoplasm of tonsillar pillars (anterior, posterior)
Neoplastic disease	1463	Malignant neoplasm of vallecula epiglottica
Neoplastic disease	1464	Malignant neoplasm of anterior aspect of epiglottis
Neoplastic disease	1465	Malignant neoplasm of junctional region of oropharynx

Neoplastic disease	1466	Malignant neoplasm of lateral wall of oropharynx
Neoplastic disease	1467	Malignant neoplasm of posterior wall of oropharynx
Neoplastic disease	1468	Malignant neoplasm of other specified sites of oropharynx
Neoplastic disease	1469	Malignant neoplasm of oropharynx unspecified site
Neoplastic disease	147	Malignant neoplasm of nasopharynx
Neoplastic disease	1470	Malignant neoplasm of superior wall of nasopharynx
Neoplastic disease	1471	Malignant neoplasm of posterior wall of nasopharynx
Neoplastic disease	1472	Malignant neoplasm of lateral wall of nasopharynx
Neoplastic disease	1473	Malignant neoplasm of anterior wall of nasopharynx
Neoplastic disease	1478	Malignant neoplasm of other specified sites of nasopharynx
Neoplastic disease	1479	Malignant neoplasm of nasopharynx unspecified site
Neoplastic disease	148	Malignant neoplasm of hypopharynx
Neoplastic disease	1480	Malignant neoplasm of postcricoid region of hypopharynx
Neoplastic disease	1481	Malignant neoplasm of pyriform sinus
Neoplastic disease	1482	Malignant neoplasm of aryepiglottic fold hypopharyngeal aspect
Neoplastic disease	1483	Malignant neoplasm of posterior hypopharyngeal wall
Neoplastic disease	1488	Malignant neoplasm of other specified sites of hypopharynx
Neoplastic disease	1489	Malignant neoplasm of hypopharynx unspecified site
Neoplastic disease	149	Malignant neoplasm of other and ill-defined sites within the lip oral cavity and pharynx
Neoplastic disease	1490	Malignant neoplasm of pharynx unspecified
Neoplastic disease	1491	Malignant neoplasm of Waldeyer's ring
Neoplastic disease	1498	Malignant neoplasm of other sites within the lip and oral cavity
Neoplastic disease	1499	Malignant neoplasm of ill-defined sites within the lip and oral cavity
Neoplastic disease	150	Malignant neoplasm of esophagus
Neoplastic disease	1500	Malignant neoplasm of cervical esophagus
Neoplastic disease	1501	Malignant neoplasm of thoracic esophagus
Neoplastic disease	1502	Malignant neoplasm of abdominal esophagus
Neoplastic disease	1503	Malignant neoplasm of upper third of esophagus
Neoplastic disease	1504	Malignant neoplasm of middle third of esophagus
Neoplastic disease	1505	Malignant neoplasm of lower third of esophagus
Neoplastic disease	1508	Malignant neoplasm of other specified part of esophagus
Neoplastic disease	1509	Malignant neoplasm of esophagus unspecified site
Neoplastic disease	151	Malignant neoplasm of stomach
Neoplastic disease	1510	Malignant neoplasm of cardia
Neoplastic disease	1511	Malignant neoplasm of pylorus
Neoplastic disease	1512	Malignant neoplasm of pyloric antrum
Neoplastic disease	1513	Malignant neoplasm of fundus of stomach
Neoplastic disease	1514	Malignant neoplasm of body of stomach
Neoplastic disease	1515	Malignant neoplasm of lesser curvature of stomach unspecified
Neoplastic disease	1516	Malignant neoplasm of greater curvature of stomach unspecified
Neoplastic disease	1518	Malignant neoplasm of other specified sites of stomach
Neoplastic disease	1519	Malignant neoplasm of stomach unspecified site
Neoplastic disease	152	Malignant neoplasm of small intestine including duodenum
Neoplastic disease	1520	Malignant neoplasm of duodenum
Neoplastic disease	1521	Malignant neoplasm of jejunum
Neoplastic disease	1522	Malignant neoplasm of ileum
Neoplastic disease	1523	Malignant neoplasm of Meckel's diverticulum
Neoplastic disease	1528	Malignant neoplasm of other specified sites of small intestine
Neoplastic disease	1529	Malignant neoplasm of small intestine unspecified site
Neoplastic disease	153	Malignant neoplasm of colon
Neoplastic disease	1530	Malignant neoplasm of hepatic flexure
Neoplastic disease	1531	Malignant neoplasm of transverse colon
Neoplastic disease	1532	Malignant neoplasm of descending colon
Neoplastic disease	1533	Malignant neoplasm of sigmoid colon
Neoplastic disease	1534	Malignant neoplasm of cecum

Neoplastic disease	1535	Malignant neoplasm of appendix vermiformis
Neoplastic disease	1536	Malignant neoplasm of ascending colon
Neoplastic disease	1537	Malignant neoplasm of splenic flexure
Neoplastic disease	1538	Malignant neoplasm of other specified sites of large intestine
Neoplastic disease	1539	Malignant neoplasm of colon unspecified site
Neoplastic disease	154	Malignant neoplasm of rectum rectosigmoid junction and anus
Neoplastic disease	1540	Malignant neoplasm of rectosigmoid junction
Neoplastic disease	1541	Malignant neoplasm of rectum
Neoplastic disease	1542	Malignant neoplasm of anal canal
Neoplastic disease	1543	Malignant neoplasm of anus unspecified site
Neoplastic disease	1548	Malignant neoplasm of other sites of rectum rectosigmoid junction and anus
Neoplastic disease	155	Malignant neoplasm of liver and intrahepatic bile ducts
Neoplastic disease	1550	Malignant neoplasm of liver primary
Neoplastic disease	1551	Malignant neoplasm of intrahepatic bile ducts
Neoplastic disease	1552	Malignant neoplasm of liver not specified as primary or secondary
Neoplastic disease	156	Malignant neoplasm of gallbladder and extrahepatic bile ducts
Neoplastic disease	1560	Malignant neoplasm of gallbladder
Neoplastic disease	1561	Malignant neoplasm of extrahepatic bile ducts
Neoplastic disease	1562	Malignant neoplasm of ampulla of Vater
Neoplastic disease	1568	Malignant neoplasm of other specified sites of gallbladder and extrahepatic bile ducts
Neoplastic disease	1569	Malignant neoplasm of biliary tract part unspecified site
Neoplastic disease	157	Malignant neoplasm of pancreas
Neoplastic disease	1570	Malignant neoplasm of head of pancreas
Neoplastic disease	1571	Malignant neoplasm of body of pancreas
Neoplastic disease	1572	Malignant neoplasm of tail of pancreas
Neoplastic disease	1573	Malignant neoplasm of pancreatic duct
Neoplastic disease	1574	Malignant neoplasm of islets of Langerhans
Neoplastic disease	1578	Malignant neoplasm of other specified sites of pancreas
Neoplastic disease	1579	Malignant neoplasm of pancreas part unspecified
Neoplastic disease	158	Malignant neoplasm of retroperitoneum and peritoneum
Neoplastic disease	1580	Malignant neoplasm of retroperitoneum
Neoplastic disease	1588	Malignant neoplasm of specified parts of peritoneum
Neoplastic disease	1589	Malignant neoplasm of peritoneum unspecified
Neoplastic disease	159	Malignant neoplasm of other and ill-defined sites within the digestive organs and peritoneum
Neoplastic disease	1590	Malignant neoplasm of intestinal tract part unspecified
Neoplastic disease	1591	Malignant neoplasm of spleen not elsewhere classified
Neoplastic disease	1598	Malignant neoplasm of other sites of digestive system and intra-abdominal organs
Neoplastic disease	1599	Malignant neoplasm of ill-defined sites within the digestive organs and peritoneum
Neoplastic disease	160	Malignant neoplasm of nasal cavities middle ear and accessory sinuses
Neoplastic disease	1600	Malignant neoplasm of nasal cavities
Neoplastic disease	1601	Malignant neoplasm of auditory tube middle ear and mastoid air cells
Neoplastic disease	1602	Malignant neoplasm of maxillary sinus
Neoplastic disease	1603	Malignant neoplasm of ethmoidal sinus
Neoplastic disease	1604	Malignant neoplasm of frontal sinus
Neoplastic disease	1605	Malignant neoplasm of sphenoidal sinus
Neoplastic disease	1608	Malignant neoplasm of other accessory sinuses
Neoplastic disease	1609	Malignant neoplasm of accessory sinus unspecified
Neoplastic disease	161	Malignant neoplasm of larynx
Neoplastic disease	1610	Malignant neoplasm of glottis
Neoplastic disease	1611	Malignant neoplasm of supraglottis
Neoplastic disease	1612	Malignant neoplasm of subglottis

Neoplastic disease	1613	Malignant neoplasm of laryngeal cartilages
Neoplastic disease	1618	Malignant neoplasm of other specified sites of larynx
Neoplastic disease	1619	Malignant neoplasm of larynx unspecified
Neoplastic disease	162	Malignant neoplasm of trachea bronchus and lung
Neoplastic disease	1620	Malignant neoplasm of trachea
Neoplastic disease	1622	Malignant neoplasm of main bronchus
Neoplastic disease	1623	Malignant neoplasm of upper lobe bronchus or lung
Neoplastic disease	1624	Malignant neoplasm of middle lobe bronchus or lung
Neoplastic disease	1625	Malignant neoplasm of lower lobe bronchus or lung
Neoplastic disease	1628	Malignant neoplasm of other parts of bronchus or lung
Neoplastic disease	1629	Malignant neoplasm of bronchus and lung unspecified
Neoplastic disease	163	Malignant neoplasm of pleura
Neoplastic disease	1630	Malignant neoplasm of parietal pleura
Neoplastic disease	1631	Malignant neoplasm of visceral pleura
Neoplastic disease	1638	Malignant neoplasm of other specified sites of pleura
Neoplastic disease	1639	Malignant neoplasm of pleura unspecified
Neoplastic disease	164	Malignant neoplasm of thymus heart and mediastinum
Neoplastic disease	1640	Malignant neoplasm of thymus
Neoplastic disease	1641	Malignant neoplasm of heart
Neoplastic disease	1642	Malignant neoplasm of anterior mediastinum
Neoplastic disease	1643	Malignant neoplasm of posterior mediastinum
Neoplastic disease	1648	Malignant neoplasm of other parts of mediastinum
Neoplastic disease	1649	Malignant neoplasm of mediastinum part unspecified
Neoplastic disease	165	Malignant neoplasm of other and ill-defined sites within the respiratory system and intrathoracic organs
Neoplastic disease	1650	Malignant neoplasm of upper respiratory tract part unspecified
Neoplastic disease	1658	Malignant neoplasm of other sites within the respiratory system and intrathoracic organs
Neoplastic disease	1659	Malignant neoplasm of ill-defined sites within the respiratory system
Neoplastic disease	170	Malignant neoplasm of bone and articular cartilage
Neoplastic disease	1700	Malignant neoplasm of bones of skull and face except mandible
Neoplastic disease	1701	Malignant neoplasm of mandible
Neoplastic disease	1702	Malignant neoplasm of vertebral column excluding sacrum and coccyx
Neoplastic disease	1703	Malignant neoplasm of ribs sternum and clavicle
Neoplastic disease	1704	Malignant neoplasm of scapula and long bones of upper limb
Neoplastic disease	1705	Malignant neoplasm of short bones of upper limb
Neoplastic disease	1706	Malignant neoplasm of pelvic bones sacrum and coccyx
Neoplastic disease	1707	Malignant neoplasm of long bones of lower limb
Neoplastic disease	1708	Malignant neoplasm of short bones of lower limb
Neoplastic disease	1709	Malignant neoplasm of bone and articular cartilage site unspecified
Neoplastic disease	171	Malignant neoplasm of connective and other soft tissue
Neoplastic disease	1710	Malignant neoplasm of connective and other soft tissue of head face and neck
Neoplastic disease	1712	Malignant neoplasm of connective and other soft tissue of upper limb including shoulder
Neoplastic disease	1713	Malignant neoplasm of connective and other soft tissue of lower limb including hip
Neoplastic disease	1714	Malignant neoplasm of connective and other soft tissue of thorax
Neoplastic disease	1715	Malignant neoplasm of connective and other soft tissue of abdomen
Neoplastic disease	1716	Malignant neoplasm of connective and other soft tissue of pelvis
Neoplastic disease	1717	Malignant neoplasm of connective and other soft tissue of trunk unspecified
Neoplastic disease	1718	Malignant neoplasm of other specified sites of connective and other soft tissue
Neoplastic disease	1719	Malignant neoplasm of connective and other soft tissue site unspecified
Neoplastic disease	172	Malignant melanoma of skin

Neoplastic disease	1720	Malignant melanoma of skin of lip
Neoplastic disease	1721	Malignant melanoma of skin of eyelid including canthus
Neoplastic disease	1722	Malignant melanoma of skin of ear and external auditory canal
Neoplastic disease	1723	Malignant melanoma of skin of other and unspecified parts of face
Neoplastic disease	1724	Malignant melanoma of skin of scalp and neck
Neoplastic disease	1725	Malignant melanoma of skin of trunk except scrotum
Neoplastic disease	1726	Malignant melanoma of skin of upper limb including shoulder
Neoplastic disease	1727	Malignant melanoma of skin of lower limb including hip
Neoplastic disease	1728	Malignant melanoma of other specified sites of skin
Neoplastic disease	1729	Melanoma of skin site unspecified
Neoplastic disease	173	Other and unspecified malignant neoplasm of skin
Neoplastic disease	1730	Other and unspecified malignant neoplasm of skin of lip
Neoplastic disease	17300	Unspecified malignant neoplasm of skin of lip
Neoplastic disease	17301	Basal cell carcinoma of skin of lip
Neoplastic disease	17302	Squamous cell carcinoma of skin of lip
Neoplastic disease	17309	Other specified malignant neoplasm of skin of lip
Neoplastic disease	1731	Other and unspecified malignant neoplasm of skin of eyelid including canthus
Neoplastic disease	17310	Unspecified malignant neoplasm of eyelid including canthus
Neoplastic disease	17311	Basal cell carcinoma of eyelid including canthus
Neoplastic disease	17312	Squamous cell carcinoma of eyelid including canthus
Neoplastic disease	17319	Other specified malignant neoplasm of eyelid including canthus
Neoplastic disease	1732	Other and unspecified malignant neoplasm of skin of ear and external auditory canal
Neoplastic disease	17320	Unspecified malignant neoplasm of skin of ear and external auditory canal
Neoplastic disease	17321	Basal cell carcinoma of skin of ear and external auditory canal
Neoplastic disease	17322	Squamous cell carcinoma of skin of ear and external auditory canal
Neoplastic disease	17329	Other specified malignant neoplasm of skin of ear and external auditory canal
Neoplastic disease	1733	Other and unspecified malignant neoplasm of skin of other and unspecified parts of face
Neoplastic disease	17330	Unspecified malignant neoplasm of skin of other and unspecified parts of face
Neoplastic disease	17331	Basal cell carcinoma of skin of other and unspecified parts of face
Neoplastic disease	17332	Squamous cell carcinoma of skin of other and unspecified parts of face
Neoplastic disease	17339	Other specified malignant neoplasm of skin of other and unspecified parts of face
Neoplastic disease	1734	Other and unspecified malignant neoplasm of scalp and skin of neck
Neoplastic disease	17340	Unspecified malignant neoplasm of scalp and skin of neck
Neoplastic disease	17341	Basal cell carcinoma of scalp and skin of neck
Neoplastic disease	17342	Squamous cell carcinoma of scalp and skin of neck

Neoplastic disease	17349	Other specified malignant neoplasm of scalp and skin of neck
Neoplastic disease	1735	Other and unspecified malignant neoplasm of skin of trunk except scrotum
Neoplastic disease	17350	Unspecified malignant neoplasm of skin of trunk except scrotum
Neoplastic disease	17351	Basal cell carcinoma of skin of trunk except scrotum
Neoplastic disease	17352	Squamous cell carcinoma of skin of trunk except scrotum
Neoplastic disease	17359	Other specified malignant neoplasm of skin of trunk except scrotum
Neoplastic disease	1736	Other and unspecified malignant neoplasm of skin of upper limb including shoulder
Neoplastic disease	17360	Unspecified malignant neoplasm of skin of upper limb including shoulder
Neoplastic disease	17361	Basal cell carcinoma of skin of upper limb including shoulder
Neoplastic disease	17362	Squamous cell carcinoma of skin of upper limb including shoulder
Neoplastic disease	17369	Other specified malignant neoplasm of skin of upper limb including shoulder
Neoplastic disease	1737	Other and unspecified malignant neoplasm of skin of lower limb including hip
Neoplastic disease	17370	Unspecified malignant neoplasm of skin of lower limb including hip
Neoplastic disease	17371	Basal cell carcinoma of skin of lower limb including hip
Neoplastic disease	17372	Squamous cell carcinoma of skin of lower limb including hip
Neoplastic disease	17379	Other specified malignant neoplasm of skin of lower limb including hip
Neoplastic disease	1738	Other and unspecified malignant neoplasm of other specified sites of skin
Neoplastic disease	17380	Unspecified malignant neoplasm of other specified sites of skin
Neoplastic disease	17381	Basal cell carcinoma of other specified sites of skin
Neoplastic disease	17382	Squamous cell carcinoma of other specified sites of skin
Neoplastic disease	17389	Other specified malignant neoplasm of other specified sites of skin
Neoplastic disease	1739	Other and unspecified malignant neoplasm of skin site unspecified
Neoplastic disease	17390	Unspecified malignant neoplasm of skin site unspecified
Neoplastic disease	17391	Basal cell carcinoma of skin site unspecified
Neoplastic disease	17392	Squamous cell carcinoma of skin site unspecified
Neoplastic disease	17399	Other specified malignant neoplasm of skin site unspecified
Neoplastic disease	174	Malignant neoplasm of female breast
Neoplastic disease	1740	Malignant neoplasm of nipple and areola of female breast
Neoplastic disease	1741	Malignant neoplasm of central portion of female breast
Neoplastic disease	1742	Malignant neoplasm of upper-inner quadrant of female breast
Neoplastic disease	1743	Malignant neoplasm of lower-inner quadrant of female breast
Neoplastic disease	1744	Malignant neoplasm of upper-outer quadrant of female breast

Neoplastic disease	1745	Malignant neoplasm of lower-outer quadrant of female breast
Neoplastic disease	1746	Malignant neoplasm of axillary tail of female breast
Neoplastic disease	1748	Malignant neoplasm of other specified sites of female breast
Neoplastic disease	1749	Malignant neoplasm of breast (female) unspecified site
Neoplastic disease	175	Malignant neoplasm of male breast
Neoplastic disease	1750	Malignant neoplasm of nipple and areola of male breast
Neoplastic disease	1759	Malignant neoplasm of other and unspecified sites of male breast
Neoplastic disease	176	Kaposi's sarcoma
Neoplastic disease	1760	Kaposi's sarcoma skin
Neoplastic disease	1761	Kaposi's sarcoma soft tissue
Neoplastic disease	1762	Kaposi's sarcoma palate
Neoplastic disease	1763	Kaposi's sarcoma gastrointestinal sites
Neoplastic disease	1764	Kaposi's sarcoma lung
Neoplastic disease	1765	Kaposi's sarcoma lymph nodes
Neoplastic disease	1768	Kaposi's sarcoma other specified sites
Neoplastic disease	1769	Kaposi's sarcoma unspecified site
Neoplastic disease	179	Malignant neoplasm of uterus-part unspecified
Neoplastic disease	180	Malignant neoplasm of cervix uteri
Neoplastic disease	1800	Malignant neoplasm of endocervix
Neoplastic disease	1801	Malignant neoplasm of exocervix
Neoplastic disease	1808	Malignant neoplasm of other specified sites of cervix
Neoplastic disease	1809	Malignant neoplasm of cervix uteri unspecified site
Neoplastic disease	181	Malignant neoplasm of placenta
Neoplastic disease	182	Malignant neoplasm of body of uterus
Neoplastic disease	1820	Malignant neoplasm of corpus uteri except isthmus
Neoplastic disease	1821	Malignant neoplasm of isthmus
Neoplastic disease	1828	Malignant neoplasm of other specified sites of body of uterus
Neoplastic disease	183	Malignant neoplasm of ovary and other uterine adnexa
Neoplastic disease	1830	Malignant neoplasm of ovary
Neoplastic disease	1832	Malignant neoplasm of fallopian tube
Neoplastic disease	1833	Malignant neoplasm of broad ligament of uterus
Neoplastic disease	1834	Malignant neoplasm of parametrium
Neoplastic disease	1835	Malignant neoplasm of round ligament of uterus
Neoplastic disease	1838	Malignant neoplasm of other specified sites of uterine adnexa
Neoplastic disease	1839	Malignant neoplasm of uterine adnexa unspecified site
Neoplastic disease	184	Malignant neoplasm of other and unspecified female genital organs
Neoplastic disease	1840	Malignant neoplasm of vagina
Neoplastic disease	1841	Malignant neoplasm of labia majora
Neoplastic disease	1842	Malignant neoplasm of labia minora
Neoplastic disease	1843	Malignant neoplasm of clitoris
Neoplastic disease	1844	Malignant neoplasm of vulva unspecified site
Neoplastic disease	1848	Malignant neoplasm of other specified sites of female genital organs
Neoplastic disease	1849	Malignant neoplasm of female genital organ site unspecified
Neoplastic disease	185	Malignant neoplasm of prostate
Neoplastic disease	186	Malignant neoplasm of testis
Neoplastic disease	1860	Malignant neoplasm of undescended testis
Neoplastic disease	1869	Malignant neoplasm of other and unspecified testis
Neoplastic disease	187	Malignant neoplasm of penis and other male genital organs
Neoplastic disease	1871	Malignant neoplasm of prepuce
Neoplastic disease	1872	Malignant neoplasm of glans penis
Neoplastic disease	1873	Malignant neoplasm of body of penis
Neoplastic disease	1874	Malignant neoplasm of penis part unspecified
Neoplastic disease	1875	Malignant neoplasm of epididymis
Neoplastic disease	1876	Malignant neoplasm of spermatic cord
Neoplastic disease	1877	Malignant neoplasm of scrotum
Neoplastic disease	1878	Malignant neoplasm of other specified sites of male genital organs

Neoplastic disease	1879	Malignant neoplasm of male genital organ site unspecified
Neoplastic disease	188	Malignant neoplasm of bladder
Neoplastic disease	1880	Malignant neoplasm of trigone of urinary bladder
Neoplastic disease	1881	Malignant neoplasm of dome of urinary bladder
Neoplastic disease	1882	Malignant neoplasm of lateral wall of urinary bladder
Neoplastic disease	1883	Malignant neoplasm of anterior wall of urinary bladder
Neoplastic disease	1884	Malignant neoplasm of posterior wall of urinary bladder
Neoplastic disease	1885	Malignant neoplasm of bladder neck
Neoplastic disease	1886	Malignant neoplasm of ureteric orifice
Neoplastic disease	1887	Malignant neoplasm of urachus
Neoplastic disease	1888	Malignant neoplasm of other specified sites of bladder
Neoplastic disease	1889	Malignant neoplasm of bladder part unspecified
Neoplastic disease	189	Malignant neoplasm of kidney and other and unspecified urinary organs
Neoplastic disease	1890	Malignant neoplasm of kidney except pelvis
Neoplastic disease	1891	Malignant neoplasm of renal pelvis
Neoplastic disease	1892	Malignant neoplasm of ureter
Neoplastic disease	1893	Malignant neoplasm of urethra
Neoplastic disease	1894	Malignant neoplasm of paraurethral glands
Neoplastic disease	1898	Malignant neoplasm of other specified sites of urinary organs
Neoplastic disease	1899	Malignant neoplasm of urinary organ site unspecified
Neoplastic disease	190	Malignant neoplasm of eye
Neoplastic disease	1900	Malignant neoplasm of eyeball except conjunctiva cornea retina and choroid
Neoplastic disease	1901	Malignant neoplasm of orbit
Neoplastic disease	1902	Malignant neoplasm of lacrimal gland
Neoplastic disease	1903	Malignant neoplasm of conjunctiva
Neoplastic disease	1904	Malignant neoplasm of cornea
Neoplastic disease	1905	Malignant neoplasm of retina
Neoplastic disease	1906	Malignant neoplasm of choroid
Neoplastic disease	1907	Malignant neoplasm of lacrimal duct
Neoplastic disease	1908	Malignant neoplasm of other specified sites of eye
Neoplastic disease	1909	Malignant neoplasm of eye part unspecified
Neoplastic disease	191	Malignant neoplasm of brain
Neoplastic disease	1910	Malignant neoplasm of cerebrum except lobes and ventricles
Neoplastic disease	1911	Malignant neoplasm of frontal lobe
Neoplastic disease	1912	Malignant neoplasm of temporal lobe
Neoplastic disease	1913	Malignant neoplasm of parietal lobe
Neoplastic disease	1914	Malignant neoplasm of occipital lobe
Neoplastic disease	1915	Malignant neoplasm of ventricles
Neoplastic disease	1916	Malignant neoplasm of cerebellum not otherwise specified
Neoplastic disease	1917	Malignant neoplasm of brain stem
Neoplastic disease	1918	Malignant neoplasm of other parts of brain
Neoplastic disease	1919	Malignant neoplasm of brain unspecified site
Neoplastic disease	192	Malignant neoplasm of other and unspecified parts of nervous system
Neoplastic disease	1920	Malignant neoplasm of cranial nerves
Neoplastic disease	1921	Malignant neoplasm of cerebral meninges
Neoplastic disease	1922	Malignant neoplasm of spinal cord
Neoplastic disease	1923	Malignant neoplasm of spinal meninges
Neoplastic disease	1928	Malignant neoplasm of other specified sites of nervous system
Neoplastic disease	1929	Malignant neoplasm of nervous system part unspecified
Neoplastic disease	193	Malignant neoplasm of thyroid gland
Neoplastic disease	194	Malignant neoplasm of other endocrine glands and related structures
Neoplastic disease	1940	Malignant neoplasm of adrenal gland
Neoplastic disease	1941	Malignant neoplasm of parathyroid gland
Neoplastic disease	1943	Malignant neoplasm of pituitary gland and craniopharyngeal duct
Neoplastic disease	1944	Malignant neoplasm of pineal gland

Neoplastic disease	1945	Malignant neoplasm of carotid body
Neoplastic disease	1946	Malignant neoplasm of aortic body and other paraganglia
Neoplastic disease	1948	Malignant neoplasm of other endocrine glands and related structures
Neoplastic disease	1949	Malignant neoplasm of endocrine gland site unspecified
Neoplastic disease	195	Malignant neoplasm of other and ill-defined sites
Neoplastic disease	1950	Malignant neoplasm of head face and neck
Neoplastic disease	1951	Malignant neoplasm of thorax
Neoplastic disease	1952	Malignant neoplasm of abdomen
Neoplastic disease	1953	Malignant neoplasm of pelvis
Neoplastic disease	1954	Malignant neoplasm of upper limb
Neoplastic disease	1955	Malignant neoplasm of lower limb
Neoplastic disease	1958	Malignant neoplasm of other specified sites
Neoplastic disease	196	Secondary and unspecified malignant neoplasm of lymph nodes
Neoplastic disease	1960	Secondary and unspecified malignant neoplasm of lymph nodes of head face and neck
Neoplastic disease	1961	Secondary and unspecified malignant neoplasm of intrathoracic lymph nodes
Neoplastic disease	1962	Secondary and unspecified malignant neoplasm of intra-abdominal lymph nodes
Neoplastic disease	1963	Secondary and unspecified malignant neoplasm of lymph nodes of axilla and upper limb
Neoplastic disease	1965	Secondary and unspecified malignant neoplasm of lymph nodes of inguinal region and lower limb
Neoplastic disease	1966	Secondary and unspecified malignant neoplasm of intrapelvic lymph nodes
Neoplastic disease	1968	Secondary and unspecified malignant neoplasm of lymph nodes of multiple sites
Neoplastic disease	1969	Secondary and unspecified malignant neoplasm of lymph nodes site unspecified
Neoplastic disease	197	Secondary malignant neoplasm of respiratory and digestive systems
Neoplastic disease	1970	Secondary malignant neoplasm of lung
Neoplastic disease	1971	Secondary malignant neoplasm of mediastinum
Neoplastic disease	1972	Secondary malignant neoplasm of pleura
Neoplastic disease	1973	Secondary malignant neoplasm of other respiratory organs
Neoplastic disease	1974	Secondary malignant neoplasm of small intestine including duodenum
Neoplastic disease	1975	Secondary malignant neoplasm of large intestine and rectum
Neoplastic disease	1976	Secondary malignant neoplasm of retroperitoneum and peritoneum
Neoplastic disease	1977	Malignant neoplasm of liver secondary
Neoplastic disease	1978	Secondary malignant neoplasm of other digestive organs and spleen
Neoplastic disease	198	Secondary malignant neoplasm of other specified sites
Neoplastic disease	1980	Secondary malignant neoplasm of kidney
Neoplastic disease	1981	Secondary malignant neoplasm of other urinary organs
Neoplastic disease	1982	Secondary malignant neoplasm of skin
Neoplastic disease	1983	Secondary malignant neoplasm of brain and spinal cord
Neoplastic disease	1984	Secondary malignant neoplasm of other parts of nervous system
Neoplastic disease	1985	Secondary malignant neoplasm of bone and bone marrow
Neoplastic disease	1986	Secondary malignant neoplasm of ovary
Neoplastic disease	1987	Secondary malignant neoplasm of adrenal gland
Neoplastic disease	1988	Secondary malignant neoplasm of other specified sites
Neoplastic disease	19881	Secondary malignant neoplasm of breast
Neoplastic disease	19882	Secondary malignant neoplasm of genital organs
Neoplastic disease	19889	Secondary malignant neoplasm of other specified sites
Neoplastic disease	199	Malignant neoplasm without specification of site

Neoplastic disease	1990	Disseminated malignant neoplasm
Neoplastic disease	1991	Other malignant neoplasm of unspecified site
Neoplastic disease	1992	Malignant neoplasm associated with transplanted organ
Neoplastic disease	200	Lymphosarcoma and reticulosarcoma and other specified malignant tumors of lymphatic tissue
Neoplastic disease	2000	Reticulosarcoma
Neoplastic disease	20000	Reticulosarcoma unspecified site
Neoplastic disease	20001	Reticulosarcoma involving lymph nodes of head face and neck
Neoplastic disease	20002	Reticulosarcoma involving intrathoracic lymph nodes
Neoplastic disease	20003	Reticulosarcoma involving intra-abdominal lymph nodes
Neoplastic disease	20004	Reticulosarcoma involving lymph nodes of axilla and upper limb
Neoplastic disease	20005	Reticulosarcoma involving lymph nodes of inguinal region and lower limb
Neoplastic disease	20006	Reticulosarcoma involving intrapelvic lymph nodes
Neoplastic disease	20007	Reticulosarcoma involving spleen
Neoplastic disease	20008	Reticulosarcoma involving lymph nodes of multiple sites
Neoplastic disease	2001	Lymphosarcoma
Neoplastic disease	20010	Lymphosarcoma unspecified site
Neoplastic disease	20011	Lymphosarcoma involving lymph nodes of head face and neck
Neoplastic disease	20012	Lymphosarcoma involving intrathoracic lymph nodes
Neoplastic disease	20013	Lymphosarcoma involving intra-abdominal lymph nodes
Neoplastic disease	20014	Lymphosarcoma involving lymph nodes of axilla and upper limb
Neoplastic disease	20015	Lymphosarcoma involving lymph nodes of inguinal region and lower limb
Neoplastic disease	20016	Lymphosarcoma involving intrapelvic lymph nodes
Neoplastic disease	20017	Lymphosarcoma involving spleen
Neoplastic disease	20018	Lymphosarcoma involving lymph nodes of multiple sites
Neoplastic disease	2002	Burkitt's tumor or lymphoma
Neoplastic disease	20020	Burkitt's tumor or lymphoma unspecified site
Neoplastic disease	20021	Burkitt's tumor or lymphoma involving lymph nodes of head face and neck
Neoplastic disease	20022	Burkitt's tumor or lymphoma involving intrathoracic lymph nodes
Neoplastic disease	20023	Burkitt's tumor or lymphoma involving intra-abdominal lymph nodes
Neoplastic disease	20024	Burkitt's tumor or lymphoma involving lymph nodes of axilla and upper limb
Neoplastic disease	20025	Burkitt's tumor or lymphoma involving lymph nodes of inguinal region and lower limb

Neoplastic disease	20026	Burkitt's tumor or lymphoma involving intrapelvic lymph nodes
Neoplastic disease	20027	Burkitt's tumor or lymphoma involving spleen
Neoplastic disease	20028	Burkitt's tumor or lymphoma involving lymph nodes of multiple sites
Neoplastic disease	2003	Marginal zone lymphoma
Neoplastic disease	20030	Marginal zone lymphoma unspecified site
Neoplastic disease	20031	Marginal zone lymphoma involving lymph nodes of head face and neck
Neoplastic disease	20032	Marginal zone lymphoma involving intrathoracic lymph nodes
Neoplastic disease	20033	Marginal zone lymphoma involving intra-abdominal lymph nodes
Neoplastic disease	20034	Marginal zone lymphoma involving lymph nodes of axilla and upper limb
Neoplastic disease	20035	Marginal zone lymphoma involving lymph nodes of inguinal region and lower limb
Neoplastic disease	20036	Marginal zone lymphoma involving intrapelvic lymph nodes
Neoplastic disease	20037	Marginal zone lymphoma involving spleen
Neoplastic disease	20038	Marginal zone lymphoma involving lymph nodes of multiple sites
Neoplastic disease	2004	Mantle cell lymphoma
Neoplastic disease	20040	Mantle cell lymphoma unspecified site
Neoplastic disease	20041	Mantle cell lymphoma involving lymph nodes of head face and neck
Neoplastic disease	20042	Mantle cell lymphoma involving intrathoracic lymph nodes
Neoplastic disease	20043	Mantle cell lymphoma involving intra-abdominal lymph nodes
Neoplastic disease	20044	Mantle cell lymphoma involving lymph nodes of axilla and upper limb
Neoplastic disease	20045	Mantle cell lymphoma involving lymph nodes of inguinal region and lower limb
Neoplastic disease	20046	Mantle cell lymphoma involving intrapelvic lymph nodes
Neoplastic disease	20047	Mantle cell lymphoma involving spleen
Neoplastic disease	20048	Mantle cell lymphoma involving lymph nodes of multiple sites
Neoplastic disease	2005	Primary central nervous system lymphoma
Neoplastic disease	20050	Primary central nervous system lymphoma unspecified site
Neoplastic disease	20051	Primary central nervous system lymphoma involving lymph nodes of head face and neck
Neoplastic disease	20052	Primary central nervous system lymphoma involving intrathoracic lymph nodes
Neoplastic disease	20053	Primary central nervous system lymphoma involving intra-abdominal lymph nodes
Neoplastic disease	20054	Primary central nervous system lymphoma involving lymph nodes of axilla and upper limb
Neoplastic disease	20055	Primary central nervous system lymphoma involving lymph nodes of inguinal region and lower limb

Neoplastic disease	2005<br>6	Primary central nervous system lymphoma involving intrapelvic lymph nodes
Neoplastic disease	2005<br>7	Primary central nervous system lymphoma involving spleen
Neoplastic disease	2005<br>8	Primary central nervous system lymphoma involving lymph nodes of multiple sites
Neoplastic disease	2006	Anaplastic large cell lymphoma
Neoplastic disease	2006<br>0	Anaplastic large cell lymphoma unspecified site
Neoplastic disease	2006<br>1	Anaplastic large cell lymphoma involving lymph nodes of head face and neck
Neoplastic disease	2006<br>2	Anaplastic large cell lymphoma involving intrathoracic lymph nodes
Neoplastic disease	2006<br>3	Anaplastic large cell lymphoma involving intra-abdominal lymph nodes
Neoplastic disease	2006<br>4	Anaplastic large cell lymphoma involving lymph nodes of axilla and upper limb
Neoplastic disease	2006<br>5	Anaplastic large cell lymphoma involving lymph nodes of inguinal region and lower limb
Neoplastic disease	2006<br>6	Anaplastic large cell lymphoma involving intrapelvic lymph nodes
Neoplastic disease	2006<br>7	Anaplastic large cell lymphoma involving spleen
Neoplastic disease	2006<br>8	Anaplastic large cell lymphoma involving lymph nodes of multiple sites
Neoplastic disease	2007	Large cell lymphoma
Neoplastic disease	2007<br>0	Large cell lymphoma unspecified site
Neoplastic disease	2007<br>1	Large cell lymphoma involving lymph nodes of head face and neck
Neoplastic disease	2007<br>2	Large cell lymphoma involving intrathoracic lymph nodes
Neoplastic disease	2007<br>3	Large cell lymphoma involving intra-abdominal lymph nodes
Neoplastic disease	2007<br>4	Large cell lymphoma involving lymph nodes of axilla and upper limb
Neoplastic disease	2007<br>5	Large cell lymphoma involving lymph nodes of inguinal region and lower limb
Neoplastic disease	2007<br>6	Large cell lymphoma involving intrapelvic lymph nodes
Neoplastic disease	2007<br>7	Large cell lymphoma involving spleen
Neoplastic disease	2007<br>8	Large cell lymphoma involving lymph nodes of multiple sites
Neoplastic disease	2008	Other named variants of lymphosarcoma and reticulosarcoma
Neoplastic disease	2008<br>0	Other named variants of lymphosarcoma and reticulosarcoma unspecified site
Neoplastic disease	2008<br>1	Other named variants of lymphosarcoma and reticulosarcoma involving lymph nodes of head face and neck
Neoplastic disease	2008<br>2	Other named variants of lymphosarcoma and reticulosarcoma involving intrathoracic lymph nodes
Neoplastic disease	2008<br>3	Other named variants of lymphosarcoma and reticulosarcoma involving intra-abdominal lymph nodes
Neoplastic disease	2008<br>4	Other named variants of lymphosarcoma and reticulosarcoma involving lymph nodes of axilla and upper limb
Neoplastic disease	2008<br>5	Other named variants of lymphosarcoma and reticulosarcoma involving lymph nodes of inguinal region and lower limb

Neoplastic disease	2008<br>6	Other named variants of lymphosarcoma and reticulosarcoma involving intrapelvic lymph nodes
Neoplastic disease	2008<br>7	Other named variants of lymphosarcoma and reticulosarcoma involving spleen
Neoplastic disease	2008<br>8	Other named variants of lymphosarcoma and reticulosarcoma involving lymph nodes of multiple sites
Neoplastic disease	201	Hodgkin's disease
Neoplastic disease	2010	Hodgkin's paragranuloma
Neoplastic disease	2010<br>0	Hodgkin's paragranuloma unspecified site
Neoplastic disease	2010<br>1	Hodgkin's paragranuloma involving lymph nodes of head face and neck
Neoplastic disease	2010<br>2	Hodgkin's paragranuloma involving intrathoracic lymph nodes
Neoplastic disease	2010<br>3	Hodgkin's paragranuloma involving intra-abdominal lymph nodes
Neoplastic disease	2010<br>4	Hodgkin's paragranuloma involving lymph nodes of axilla and upper limb
Neoplastic disease	2010<br>5	Hodgkin's paragranuloma involving lymph nodes of inguinal region and lower limb
Neoplastic disease	2010<br>6	Hodgkin's paragranuloma involving intrapelvic lymph nodes
Neoplastic disease	2010<br>7	Hodgkin's paragranuloma involving spleen
Neoplastic disease	2010<br>8	Hodgkin's paragranuloma involving lymph nodes of multiple sites
Neoplastic disease	2011	Hodgkin's granuloma
Neoplastic disease	2011<br>0	Hodgkin's granuloma unspecified site
Neoplastic disease	2011<br>1	Hodgkin's granuloma involving lymph nodes of head face and neck
Neoplastic disease	2011<br>2	Hodgkin's granuloma involving intrathoracic lymph nodes
Neoplastic disease	2011<br>3	Hodgkin's granuloma involving intra-abdominal lymph nodes
Neoplastic disease	2011<br>4	Hodgkin's granuloma involving lymph nodes of axilla and upper limb
Neoplastic disease	2011<br>5	Hodgkin's granuloma involving lymph nodes of inguinal region and lower limb
Neoplastic disease	2011<br>6	Hodgkin's granuloma involving intrapelvic lymph nodes
Neoplastic disease	2011<br>7	Hodgkin's granuloma involving spleen
Neoplastic disease	2011<br>8	Hodgkin's granuloma involving lymph nodes of multiple sites
Neoplastic disease	2012	Hodgkin's sarcoma
Neoplastic disease	2012<br>0	Hodgkin's sarcoma unspecified site
Neoplastic disease	2012<br>1	Hodgkin's sarcoma involving lymph nodes of head face and neck
Neoplastic disease	2012<br>2	Hodgkin's sarcoma involving intrathoracic lymph nodes
Neoplastic disease	2012<br>3	Hodgkin's sarcoma involving intra-abdominal lymph nodes
Neoplastic disease	2012<br>4	Hodgkin's sarcoma involving lymph nodes of axilla and upper limb

Neoplastic disease	2012 5	Hodgkin's sarcoma involving lymph nodes of inguinal region and lower limb
Neoplastic disease	2012 6	Hodgkin's sarcoma involving intrapelvic lymph nodes
Neoplastic disease	2012 7	Hodgkin's sarcoma involving spleen
Neoplastic disease	2012 8	Hodgkin's sarcoma involving lymph nodes of multiple sites
Neoplastic disease	2014	Hodgkin's disease lymphocytic-histiocytic predominance
Neoplastic disease	2014 0	Hodgkin's disease lymphocytic-histiocytic predominance unspecified site
Neoplastic disease	2014 1	Hodgkin's disease lymphocytic-histiocytic predominance involving lymph nodes of head face and neck
Neoplastic disease	2014 2	Hodgkin's disease lymphocytic-histiocytic predominance involving intrathoracic lymph nodes
Neoplastic disease	2014 3	Hodgkin's disease lymphocytic-histiocytic predominance involving intra-abdominal lymph nodes
Neoplastic disease	2014 4	Hodgkin's disease lymphocytic-histiocytic predominance involving lymph nodes of axilla and upper limb
Neoplastic disease	2014 5	Hodgkin's disease lymphocytic-histiocytic predominance involving lymph nodes of inguinal region and lower limb
Neoplastic disease	2014 6	Hodgkin's disease lymphocytic-histiocytic predominance involving intrapelvic lymph nodes
Neoplastic disease	2014 7	Hodgkin's disease lymphocytic-histiocytic predominance involving spleen
Neoplastic disease	2014 8	Hodgkin's disease lymphocytic-histiocytic predominance involving lymph nodes of multiple sites
Neoplastic disease	2015	Hodgkin's disease nodular sclerosis
Neoplastic disease	2015 0	Hodgkin's disease nodular sclerosis unspecified site
Neoplastic disease	2015 1	Hodgkin's disease nodular sclerosis involving lymph nodes of head face and neck
Neoplastic disease	2015 2	Hodgkin's disease nodular sclerosis involving intrathoracic lymph nodes
Neoplastic disease	2015 3	Hodgkin's disease nodular sclerosis involving intra-abdominal lymph nodes
Neoplastic disease	2015 4	Hodgkin's disease nodular sclerosis involving lymph nodes of axilla and upper limb
Neoplastic disease	2015 5	Hodgkin's disease nodular sclerosis involving lymph nodes of inguinal region and lower limb
Neoplastic disease	2015 6	Hodgkin's disease nodular sclerosis involving intrapelvic lymph nodes
Neoplastic disease	2015 7	Hodgkin's disease nodular sclerosis involving spleen
Neoplastic disease	2015 8	Hodgkin's disease nodular sclerosis involving lymph nodes of multiple sites
Neoplastic disease	2016	Hodgkin's disease mixed cellularity
Neoplastic disease	2016 0	Hodgkin's disease mixed cellularity unspecified site
Neoplastic disease	2016 1	Hodgkin's disease mixed cellularity involving lymph nodes of head face and neck
Neoplastic disease	2016 2	Hodgkin's disease mixed cellularity involving intrathoracic lymph nodes
Neoplastic disease	2016 3	Hodgkin's disease mixed cellularity involving intra-abdominal lymph nodes
Neoplastic disease	2016 4	Hodgkin's disease mixed cellularity involving lymph nodes of axilla and upper limb

Neoplastic disease	2016 5	Hodgkin's disease mixed cellularity involving lymph nodes of inguinal region and lower limb
Neoplastic disease	2016 6	Hodgkin's disease mixed cellularity involving intrapelvic lymph nodes
Neoplastic disease	2016 7	Hodgkin's disease mixed cellularity involving spleen
Neoplastic disease	2016 8	Hodgkin's disease mixed cellularity involving lymph nodes of multiple sites
Neoplastic disease	2017	Hodgkin's disease lymphocytic depletion
Neoplastic disease	2017 0	Hodgkin's disease lymphocytic depletion unspecified site
Neoplastic disease	2017 1	Hodgkin's disease lymphocytic depletion involving lymph nodes of head face and neck
Neoplastic disease	2017 2	Hodgkin's disease lymphocytic depletion involving intrathoracic lymph nodes
Neoplastic disease	2017 3	Hodgkin's disease lymphocytic depletion involving intra-abdominal lymph nodes
Neoplastic disease	2017 4	Hodgkin's disease lymphocytic depletion involving lymph nodes of axilla and upper limb
Neoplastic disease	2017 5	Hodgkin's disease lymphocytic depletion involving lymph nodes of inguinal region and lower limb
Neoplastic disease	2017 6	Hodgkin's disease lymphocytic depletion involving intrapelvic lymph nodes
Neoplastic disease	2017 7	Hodgkin's disease lymphocytic depletion involving spleen
Neoplastic disease	2017 8	Hodgkin's disease lymphocytic depletion involving lymph nodes of multiple sites
Neoplastic disease	2019	Hodgkin's disease unspecified type
Neoplastic disease	2019 0	Hodgkin's disease unspecified type unspecified site
Neoplastic disease	2019 1	Hodgkin's disease unspecified type involving lymph nodes of head face and neck
Neoplastic disease	2019 2	Hodgkin's disease unspecified type involving intrathoracic lymph nodes
Neoplastic disease	2019 3	Hodgkin's disease unspecified type involving intra-abdominal lymph nodes
Neoplastic disease	2019 4	Hodgkin's disease unspecified type involving lymph nodes of axilla and upper limb
Neoplastic disease	2019 5	Hodgkin's disease unspecified type involving lymph nodes of inguinal region and lower limb
Neoplastic disease	2019 6	Hodgkin's disease unspecified type involving intrapelvic lymph nodes
Neoplastic disease	2019 7	Hodgkin's disease unspecified type involving spleen
Neoplastic disease	2019 8	Hodgkin's disease unspecified type involving lymph nodes of multiple sites
Neoplastic disease	202	Other malignant neoplasms of lymphoid and histiocytic tissue
Neoplastic disease	2020	Nodular lymphoma
Neoplastic disease	2020 0	Nodular lymphoma unspecified site
Neoplastic disease	2020 1	Nodular lymphoma involving lymph nodes of head face and neck
Neoplastic disease	2020 2	Nodular lymphoma involving intrathoracic lymph nodes
Neoplastic disease	2020 3	Nodular lymphoma involving intra-abdominal lymph nodes

Neoplastic disease	20204	Nodular lymphoma involving lymph nodes of axilla and upper limb
Neoplastic disease	20205	Nodular lymphoma involving lymph nodes of inguinal region and lower limb
Neoplastic disease	20206	Nodular lymphoma involving intrapelvic lymph nodes
Neoplastic disease	20207	Nodular lymphoma involving spleen
Neoplastic disease	20208	Nodular lymphoma involving lymph nodes of multiple sites
Neoplastic disease	2021	Mycosis fungoides
Neoplastic disease	20210	Mycosis fungoides unspecified site
Neoplastic disease	20211	Mycosis fungoides involving lymph nodes of head face and neck
Neoplastic disease	20212	Mycosis fungoides involving intrathoracic lymph nodes
Neoplastic disease	20213	Mycosis fungoides involving intra-abdominal lymph nodes
Neoplastic disease	20214	Mycosis fungoides involving lymph nodes of axilla and upper limb
Neoplastic disease	20215	Mycosis fungoides involving lymph nodes of inguinal region and lower limb
Neoplastic disease	20216	Mycosis fungoides involving intrapelvic lymph nodes
Neoplastic disease	20217	Mycosis fungoides involving spleen
Neoplastic disease	20218	Mycosis fungoides involving lymph nodes of multiple sites
Neoplastic disease	2022	Sezary's disease
Neoplastic disease	20220	Sezary's disease unspecified site
Neoplastic disease	20221	Sezary's disease involving lymph nodes of head face and neck
Neoplastic disease	20222	Sezary's disease involving intrathoracic lymph nodes
Neoplastic disease	20223	Sezary's disease involving intra-abdominal lymph nodes
Neoplastic disease	20224	Sezary's disease involving lymph nodes of axilla and upper limb
Neoplastic disease	20225	Sezary's disease involving lymph nodes of inguinal region and lower limb
Neoplastic disease	20226	Sezary's disease involving intrapelvic lymph nodes
Neoplastic disease	20227	Sezary's disease involving spleen
Neoplastic disease	20228	Sezary's disease involving lymph nodes of multiple sites
Neoplastic disease	2023	Malignant histiocytosis
Neoplastic disease	20230	Malignant histiocytosis unspecified site
Neoplastic disease	20231	Malignant histiocytosis involving lymph nodes of head face and neck
Neoplastic disease	20232	Malignant histiocytosis involving intrathoracic lymph nodes
Neoplastic disease	20233	Malignant histiocytosis involving intra-abdominal lymph nodes

Neoplastic disease	2023<br>4	Malignant histiocytosis involving lymph nodes of axilla and upper limb
Neoplastic disease	2023<br>5	Malignant histiocytosis involving lymph nodes of inguinal region and lower limb
Neoplastic disease	2023<br>6	Malignant histiocytosis involving intrapelvic lymph nodes
Neoplastic disease	2023<br>7	Malignant histiocytosis involving spleen
Neoplastic disease	2023<br>8	Malignant histiocytosis involving lymph nodes of multiple sites
Neoplastic disease	2024	Leukemic reticuloendotheliosis
Neoplastic disease	2024<br>0	Leukemic reticuloendotheliosis unspecified site
Neoplastic disease	2024<br>1	Leukemic reticuloendotheliosis involving lymph nodes of head face and neck
Neoplastic disease	2024<br>2	Leukemic reticuloendotheliosis involving intrathoracic lymph nodes
Neoplastic disease	2024<br>3	Leukemic reticuloendotheliosis involving intra-abdominal lymph nodes
Neoplastic disease	2024<br>4	Leukemic reticuloendotheliosis involving lymph nodes of axilla and upper arm
Neoplastic disease	2024<br>5	Leukemic reticuloendotheliosis involving lymph nodes of inguinal region and lower limb
Neoplastic disease	2024<br>6	Leukemic reticuloendotheliosis involving intrapelvic lymph nodes
Neoplastic disease	2024<br>7	Leukemic reticuloendotheliosis involving spleen
Neoplastic disease	2024<br>8	Leukemic reticuloendotheliosis involving lymph nodes of multiple sites
Neoplastic disease	2025	Letterer-Siwe disease
Neoplastic disease	2025<br>0	Letterer-Siwe disease unspecified site
Neoplastic disease	2025<br>1	Letterer-Siwe disease involving lymph nodes of head face and neck
Neoplastic disease	2025<br>2	Letterer-Siwe disease involving intrathoracic lymph nodes
Neoplastic disease	2025<br>3	Letterer-Siwe disease involving intra-abdominal lymph nodes
Neoplastic disease	2025<br>4	Letterer-Siwe disease involving lymph nodes of axilla and upper limb
Neoplastic disease	2025<br>5	Letterer-Siwe disease involving lymph nodes of inguinal region and lower limb
Neoplastic disease	2025<br>6	Letterer-Siwe disease involving intrapelvic lymph nodes
Neoplastic disease	2025<br>7	Letterer-Siwe disease involving spleen
Neoplastic disease	2025<br>8	Letterer-Siwe disease involving lymph nodes of multiple sites
Neoplastic disease	2026	Malignant mast cell tumors
Neoplastic disease	2026<br>0	Malignant mast cell tumors unspecified site
Neoplastic disease	2026<br>1	Malignant mast cell tumors involving lymph nodes of head face and neck
Neoplastic disease	2026<br>2	Malignant mast cell tumors involving intrathoracic lymph nodes
Neoplastic disease	2026<br>3	Malignant mast cell tumors involving intra-abdominal lymph nodes

Neoplastic disease	20264	Malignant mast cell tumors involving lymph nodes of axilla and upper limb
Neoplastic disease	20265	Malignant mast cell tumors involving lymph nodes of inguinal region and lower limb
Neoplastic disease	20266	Malignant mast cell tumors involving intrapelvic lymph nodes
Neoplastic disease	20267	Malignant mast cell tumors involving spleen
Neoplastic disease	20268	Malignant mast cell tumors involving lymph nodes of multiple sites
Neoplastic disease	2027	Peripheral T-cell lymphoma
Neoplastic disease	20270	Peripheral T-cell lymphoma unspecified site
Neoplastic disease	20271	Peripheral T-cell lymphoma involving lymph nodes of head face and neck
Neoplastic disease	20272	Peripheral T-cell lymphoma involving intrathoracic lymph nodes
Neoplastic disease	20273	Peripheral T-cell lymphoma involving intra-abdominal lymph nodes
Neoplastic disease	20274	Peripheral T-cell lymphoma involving lymph nodes of axilla and upper limb
Neoplastic disease	20275	Peripheral T-cell lymphoma involving lymph nodes of inguinal region and lower limb
Neoplastic disease	20276	Peripheral T-cell lymphoma involving intrapelvic lymph nodes
Neoplastic disease	20277	Peripheral T-cell lymphoma involving spleen
Neoplastic disease	20278	Peripheral T-cell lymphoma involving lymph nodes of multiple sites
Neoplastic disease	2028	Other malignant lymphomas
Neoplastic disease	20280	Other malignant lymphomas unspecified site
Neoplastic disease	20281	Other malignant lymphomas involving lymph nodes of head face and neck
Neoplastic disease	20282	Other malignant lymphomas involving intrathoracic lymph nodes
Neoplastic disease	20283	Other malignant lymphomas involving intra-abdominal lymph nodes
Neoplastic disease	20284	Other malignant lymphomas involving lymph nodes of axilla and upper limb
Neoplastic disease	20285	Other malignant lymphomas involving lymph nodes of inguinal region and lower limb
Neoplastic disease	20286	Other malignant lymphomas involving intrapelvic lymph nodes
Neoplastic disease	20287	Other malignant lymphomas involving spleen
Neoplastic disease	20288	Other malignant lymphomas involving lymph nodes of multiple sites
Neoplastic disease	2029	Other and unspecified malignant neoplasms of lymphoid and histiocytic tissue
Neoplastic disease	20290	Other and unspecified malignant neoplasms of lymphoid and histiocytic tissue unspecified site
Neoplastic disease	20291	Other and unspecified malignant neoplasms of lymphoid and histiocytic tissue involving lymph nodes of head face and neck
Neoplastic disease	20292	Other and unspecified malignant neoplasms of lymphoid and histiocytic tissue involving intrathoracic lymph nodes

Neoplastic disease	20293	Other and unspecified malignant neoplasms of lymphoid and histiocytic tissue involving intra-abdominal lymph nodes
Neoplastic disease	20294	Other and unspecified malignant neoplasms of lymphoid and histiocytic tissue involving lymph nodes of axilla and upper limb
Neoplastic disease	20295	Other and unspecified malignant neoplasms of lymphoid and histiocytic tissue involving lymph nodes of inguinal region and lower limb
Neoplastic disease	20296	Other and unspecified malignant neoplasms of lymphoid and histiocytic tissue involving intrapelvic lymph nodes
Neoplastic disease	20297	Other and unspecified malignant neoplasms of lymphoid and histiocytic tissue involving spleen
Neoplastic disease	20298	Other and unspecified malignant neoplasms of lymphoid and histiocytic tissue involving lymph nodes of multiple sites
Neoplastic disease	203	Multiple myeloma and immunoproliferative neoplasms
Neoplastic disease	2030	Multiple myeloma
Neoplastic disease	20300	Multiple myeloma without mention of having achieved remission
Neoplastic disease	20301	Multiple myeloma in remission
Neoplastic disease	20302	Multiple myeloma in relapse
Neoplastic disease	2031	Plasma cell leukemia
Neoplastic disease	20310	Plasma cell leukemia without mention of having achieved remission
Neoplastic disease	20311	Plasma cell leukemia in remission
Neoplastic disease	20312	Plasma cell leukemia in relapse
Neoplastic disease	2038	Other immunoproliferative neoplasms
Neoplastic disease	20380	Other immunoproliferative neoplasms without mention of having achieved remission
Neoplastic disease	20381	Other immunoproliferative neoplasms in remission
Neoplastic disease	20382	Other immunoproliferative neoplasms in relapse
Neoplastic disease	204	Lymphoid leukemia
Neoplastic disease	2040	Acute lymphoid leukemia
Neoplastic disease	20400	Acute lymphoid leukemia without mention of having achieved remission
Neoplastic disease	20401	Acute lymphoid leukemia in remission
Neoplastic disease	20402	Acute lymphoid leukemia in relapse
Neoplastic disease	2041	Chronic lymphoid leukemia
Neoplastic disease	20410	Chronic lymphoid leukemia without mention of having achieved remission
Neoplastic disease	20411	Chronic lymphoid leukemia in remission
Neoplastic disease	20412	Chronic lymphoid leukemia in relapse
Neoplastic disease	2042	Subacute lymphoid leukemia
Neoplastic disease	20420	Subacute lymphoid leukemia without mention of having achieved remission
Neoplastic disease	20421	Subacute lymphoid leukemia in remission
Neoplastic disease	20422	Subacute lymphoid leukemia in relapse
Neoplastic disease	2048	Other lymphoid leukemia

Neoplastic disease	20480	Other lymphoid leukemia without mention of having achieved remission
Neoplastic disease	20481	Other lymphoid leukemia in remission
Neoplastic disease	20482	Other lymphoid leukemia in relapse
Neoplastic disease	2049	Unspecified lymphoid leukemia
Neoplastic disease	20490	Unspecified lymphoid leukemia without mention of having achieved remission
Neoplastic disease	20491	Unspecified lymphoid leukemia in remission
Neoplastic disease	20492	Unspecified lymphoid leukemia in relapse
Neoplastic disease	205	Myeloid leukemia
Neoplastic disease	2050	Acute myeloid leukemia
Neoplastic disease	20500	Acute myeloid leukemia without mention of having achieved remission
Neoplastic disease	20501	Acute myeloid leukemia in remission
Neoplastic disease	20502	Acute myeloid leukemia in relapse
Neoplastic disease	2051	Chronic myeloid leukemia
Neoplastic disease	20510	Chronic myeloid leukemia without mention of having achieved remission
Neoplastic disease	20511	Chronic myeloid leukemia in remission
Neoplastic disease	20512	Chronic myeloid leukemia in relapse
Neoplastic disease	2052	Subacute myeloid leukemia
Neoplastic disease	20520	Subacute myeloid leukemia without mention of having achieved remission
Neoplastic disease	20521	Subacute myeloid leukemia in remission
Neoplastic disease	20522	Subacute myeloid leukemia in relapse
Neoplastic disease	2053	Myeloid sarcoma
Neoplastic disease	20530	Myeloid sarcoma without mention of having achieved remission
Neoplastic disease	20531	Myeloid sarcoma in remission
Neoplastic disease	20532	Myeloid sarcoma in relapse
Neoplastic disease	2058	Other myeloid leukemia
Neoplastic disease	20580	Other myeloid leukemia without mention of having achieved remission
Neoplastic disease	20581	Other myeloid leukemia in remission
Neoplastic disease	20582	Other myeloid leukemia in relapse
Neoplastic disease	2059	Unspecified myeloid leukemia
Neoplastic disease	20590	Unspecified myeloid leukemia without mention of having achieved remission
Neoplastic disease	20591	Unspecified myeloid leukemia in remission
Neoplastic disease	20592	Unspecified myeloid leukemia in relapse
Neoplastic disease	206	Monocytic leukemia

Neoplastic disease	2060	Acute monocytic leukemia
Neoplastic disease	20600	Acute monocytic leukemia without mention of having achieved remission
Neoplastic disease	20601	Acute monocytic leukemia in remission
Neoplastic disease	20602	Acute monocytic leukemia in relapse
Neoplastic disease	2061	Chronic monocytic leukemia
Neoplastic disease	20610	Chronic monocytic leukemia without mention of having achieved remission
Neoplastic disease	20611	Chronic monocytic leukemia in remission
Neoplastic disease	20612	Chronic monocytic leukemia in relapse
Neoplastic disease	2062	Subacute monocytic leukemia
Neoplastic disease	20620	Subacute monocytic leukemia without mention of having achieved remission
Neoplastic disease	20621	Subacute monocytic leukemia in remission
Neoplastic disease	20622	Subacute monocytic leukemia in relapse
Neoplastic disease	2068	Other monocytic leukemia
Neoplastic disease	20680	Other monocytic leukemia without mention of having achieved remission
Neoplastic disease	20681	Other monocytic leukemia in remission
Neoplastic disease	20682	Other monocytic leukemia in relapse
Neoplastic disease	2069	Unspecified monocytic leukemia
Neoplastic disease	20690	Unspecified monocytic leukemia without mention of having achieved remission
Neoplastic disease	20691	Unspecified monocytic leukemia in remission
Neoplastic disease	20692	Unspecified monocytic leukemia in relapse
Neoplastic disease	207	Other specified leukemia
Neoplastic disease	2070	Acute erythremia and erythroleukemia
Neoplastic disease	20700	Acute erythremia and erythroleukemia without mention of having achieved remission
Neoplastic disease	20701	Acute erythremia and erythroleukemia in remission
Neoplastic disease	20702	Acute erythremia and erythroleukemia in relapse
Neoplastic disease	2071	Chronic erythremia
Neoplastic disease	20710	Chronic erythremia without mention of having achieved remission
Neoplastic disease	20711	Chronic erythremia in remission
Neoplastic disease	20712	Chronic erythremia in relapse
Neoplastic disease	2072	Megakaryocytic leukemia
Neoplastic disease	20720	Megakaryocytic leukemia without mention of having achieved remission
Neoplastic disease	20721	Megakaryocytic leukemia in remission
Neoplastic disease	20722	Megakaryocytic leukemia in relapse

Neoplastic disease	2078	Other specified leukemia
Neoplastic disease	20780	Other specified leukemia without mention of having achieved remission
Neoplastic disease	20781	Other specified leukemia in remission
Neoplastic disease	20782	Other specified leukemia in relapse
Neoplastic disease	208	Leukemia of unspecified cell type
Neoplastic disease	2080	Acute leukemia of unspecified cell type
Neoplastic disease	20800	Acute leukemia of unspecified cell type without mention of having achieved remission
Neoplastic disease	20801	Acute leukemia of unspecified cell type in remission
Neoplastic disease	20802	Acute leukemia of unspecified cell type in relapse
Neoplastic disease	2081	Chronic leukemia of unspecified cell type
Neoplastic disease	20810	Chronic leukemia of unspecified cell type without mention of having achieved remission
Neoplastic disease	20811	Chronic leukemia of unspecified cell type in remission
Neoplastic disease	20812	Chronic leukemia of unspecified cell type in relapse
Neoplastic disease	2082	Subacute leukemia of unspecified cell type
Neoplastic disease	20820	Subacute leukemia of unspecified cell type without mention of having achieved remission
Neoplastic disease	20821	Subacute leukemia of unspecified cell type in remission
Neoplastic disease	20822	Subacute leukemia of unspecified cell type in relapse
Neoplastic disease	2088	Other leukemia of unspecified cell type
Neoplastic disease	20880	Other leukemia of unspecified cell type without mention of having achieved remission
Neoplastic disease	20881	Other leukemia of unspecified cell type in remission
Neoplastic disease	20882	Other leukemia of unspecified cell type in relapse
Neoplastic disease	2089	Unspecified leukemia
Neoplastic disease	20890	Unspecified leukemia without mention of having achieved remission
Neoplastic disease	20891	Unspecified leukemia in remission
Neoplastic disease	20892	Unspecified leukemia in relapse
Neoplastic disease	2090	Malignant carcinoid tumors of the small intestine
Neoplastic disease	20900	Malignant carcinoid tumor of the small intestine unspecified portion
Neoplastic disease	20901	Malignant carcinoid tumor of the duodenum
Neoplastic disease	20902	Malignant carcinoid tumor of the jejunum
Neoplastic disease	20903	Malignant carcinoid tumor of the ileum
Neoplastic disease	2091	Malignant carcinoid tumors of the appendix large intestine and rectum
Neoplastic disease	20910	Malignant carcinoid tumor of the large intestine unspecified portion
Neoplastic disease	20911	Malignant carcinoid tumor of the appendix

Neoplastic disease	20912	Malignant carcinoid tumor of the cecum
Neoplastic disease	20913	Malignant carcinoid tumor of the ascending colon
Neoplastic disease	20914	Malignant carcinoid tumor of the transverse colon
Neoplastic disease	20915	Malignant carcinoid tumor of the descending colon
Neoplastic disease	20916	Malignant carcinoid tumor of the sigmoid colon
Neoplastic disease	20917	Malignant carcinoid tumor of the rectum
Neoplastic disease	2092	Malignant carcinoid tumor of other and unspecified sites
Neoplastic disease	20920	Malignant carcinoid tumor of unknown primary site
Neoplastic disease	20921	Malignant carcinoid tumor of the bronchus and lung
Neoplastic disease	20922	Malignant carcinoid tumor of the thymus
Neoplastic disease	20923	Malignant carcinoid tumor of the stomach
Neoplastic disease	20924	Malignant carcinoid tumor of the kidney
Neoplastic disease	20925	Malignant carcinoid tumor of the foregut not otherwise specified
Neoplastic disease	20926	Malignant carcinoid tumor of the midgut not otherwise specified
Neoplastic disease	20927	Malignant carcinoid tumor of the hindgut not otherwise specified
Neoplastic disease	20929	Malignant carcinoid tumor of other sites
Neoplastic disease	2093	Malignant poorly differentiated neuroendocrine tumors
Neoplastic disease	20930	Malignant poorly differentiated neuroendocrine carcinoma any site
Neoplastic disease	20931	Merkel cell carcinoma of the face
Neoplastic disease	20932	Merkel cell carcinoma of the scalp and neck
Neoplastic disease	20933	Merkel cell carcinoma of the upper limb
Neoplastic disease	20934	Merkel cell carcinoma of the lower limb
Neoplastic disease	20935	Merkel cell carcinoma of the trunk
Neoplastic disease	20936	Merkel cell carcinoma of other sites
Neoplastic disease	2097	Secondary neuroendocrine tumors
Neoplastic disease	20970	Secondary neuroendocrine tumor unspecified site
Neoplastic disease	20971	Secondary neuroendocrine tumor of distant lymph nodes
Neoplastic disease	20972	Secondary neuroendocrine tumor of liver
Neoplastic disease	20973	Secondary neuroendocrine tumor of bone
Neoplastic disease	20974	Secondary neuroendocrine tumor of peritoneum

Neoplastic disease	2097<br>5	Secondary Merkel cell carcinoma
Neoplastic disease	2097<br>9	Secondary neuroendocrine tumor of other sites
Neoplastic disease	230	Carcinoma in situ of digestive organs
Neoplastic disease	2300	Carcinoma in situ of lip oral cavity and pharynx
Neoplastic disease	2301	Carcinoma in situ of esophagus
Neoplastic disease	2302	Carcinoma in situ of stomach
Neoplastic disease	2303	Carcinoma in situ of colon
Neoplastic disease	2304	Carcinoma in situ of rectum
Neoplastic disease	2305	Carcinoma in situ of anal canal
Neoplastic disease	2306	Carcinoma in situ of anus unspecified
Neoplastic disease	2307	Carcinoma in situ of other and unspecified parts of intestine
Neoplastic disease	2308	Carcinoma in situ of liver and biliary system
Neoplastic disease	2309	Carcinoma in situ of other and unspecified digestive organs
Neoplastic disease	231	Carcinoma in situ of respiratory system
Neoplastic disease	2310	Carcinoma in situ of larynx
Neoplastic disease	2311	Carcinoma in situ of trachea
Neoplastic disease	2312	Carcinoma in situ of bronchus and lung
Neoplastic disease	2318	Carcinoma in situ of other specified parts of respiratory system
Neoplastic disease	2319	Carcinoma in situ of respiratory system part unspecified
Neoplastic disease	232	Carcinoma in situ of skin
Neoplastic disease	2320	Carcinoma in situ of skin of lip
Neoplastic disease	2321	Carcinoma in situ of eyelid including canthus
Neoplastic disease	2322	Carcinoma in situ of skin of ear and external auditory canal
Neoplastic disease	2323	Carcinoma in situ of skin of other and unspecified parts of face
Neoplastic disease	2324	Carcinoma in situ of scalp and skin of neck
Neoplastic disease	2325	Carcinoma in situ of skin of trunk except scrotum
Neoplastic disease	2326	Carcinoma in situ of skin of upper limb including shoulder
Neoplastic disease	2327	Carcinoma in situ of skin of lower limb including hip
Neoplastic disease	2328	Carcinoma in situ of other specified sites of skin
Neoplastic disease	2329	Carcinoma in situ of skin site unspecified
Neoplastic disease	233	Carcinoma in situ of breast and genitourinary system
Neoplastic disease	2330	Carcinoma in situ of breast
Neoplastic disease	2331	Carcinoma in situ of cervix uteri
Neoplastic disease	2332	Carcinoma in situ of other and unspecified parts of uterus
Neoplastic disease	2333	Carcinoma in situ of other and unspecified female genital organs
Neoplastic disease	2333<br>0	Carcinoma in situ of unspecified female genital organ
Neoplastic disease	2333<br>1	Carcinoma in situ of vagina
Neoplastic disease	2333<br>2	Carcinoma in situ of vulva
Neoplastic disease	2333<br>9	Carcinoma in situ of other female genital organ
Neoplastic disease	2334	Carcinoma in situ of prostate
Neoplastic disease	2335	Carcinoma in situ of penis
Neoplastic disease	2336	Carcinoma in situ of other and unspecified male genital organs
Neoplastic disease	2337	Carcinoma in situ of bladder
Neoplastic disease	2339	Carcinoma in situ of other and unspecified urinary organs
Neoplastic disease	234	Carcinoma in situ of other and unspecified sites
Neoplastic disease	2340	Carcinoma in situ of eye
Neoplastic disease	2348	Carcinoma in situ of other specified sites
Neoplastic disease	2349	Carcinoma in situ site unspecified
Neoplastic disease	2384	Polycythemia vera
Neoplastic disease	2386	Neoplasm of uncertain behavior of plasma cells

Neoplastic disease	2387	Neoplasm of uncertain behavior of other lymphatic and hematopoietic tissues
Neoplastic disease	2387<br>1	Essential thrombocythemia
Neoplastic disease	2387<br>2	Low grade myelodysplastic syndrome lesions
Neoplastic disease	2387<br>3	High grade myelodysplastic syndrome lesions
Neoplastic disease	2387<br>4	Myelodysplastic syndrome with 5q deletion
Neoplastic disease	2387<br>5	Myelodysplastic syndrome unspecified
Neoplastic disease	2387<br>6	Myelofibrosis with myeloid metaplasia
Neoplastic disease	2387<br>7	Post-transplant lymphoproliferative disorder
Neoplastic disease	2387<br>9	Neoplasm of uncertain behavior of other lymphatic and hematopoietic tissues
Neoplastic disease	2580<br>2	Multiple endocrine neoplasia type IIa
Neoplastic disease	2580<br>3	Multiple endocrine neoplasia type IIb
Neoplastic disease	2733	Macroglobulinemia
Neoplastic disease	2898<br>3	Myelofibrosis
Neoplastic disease	3573	Polyneuropathy in malignant disease
Neoplastic disease	5118<br>1	Malignant pleural effusion
Neoplastic disease	7895<br>1	Malignant ascites
Neoplastic disease	7950<br>4	Papanicolaou smear of cervix with HGSIL
Neoplastic disease	7950<br>6	Papanicolaou smear of cervix with cytologic evidence of malignancy
Neoplastic disease	7951<br>4	Papanicolaou smear of vagina with HGSIL
Neoplastic disease	7951<br>6	Papanicolaou smear of vagina with cytologic evidence of malignancy
Neoplastic disease	7967<br>4	Papanicolaou smear of anus with HGSIL
Neoplastic disease	7967<br>6	Papanicolaou smear of anus with cytologic evidence of malignancy
Neoplastic disease	2841<br>1	Antineoplastic chemotherapy induced pancytopenia
Neoplastic disease	5280<br>1	Mucositis (ulcerative) due to antineoplastic therapy
Neoplastic disease	E930<br>7	Antineoplastic antibiotics causing adverse effects in therapeutic use
Neoplastic disease	E933<br>1	Antineoplastic and immunosuppressive drugs causing adverse effects in therapeutic use
Neoplastic disease	V581	Encounter for antineoplastic chemotherapy and immunotherapy
Neoplastic disease	V581<br>1	Encounter for antineoplastic chemotherapy
Neoplastic disease	V581<br>2	Encounter for antineoplastic immunotherapy
Liver disease history	0063	Amebic liver abscess
Liver disease history	070	Viral hepatitis

Liver disease history	0701	Viral hepatitis a without hepatic coma
Liver disease history	0703	Viral hepatitis b without mention of hepatic coma
Liver disease history	07030	Viral hepatitis b without hepatic coma acute or unspecified without hepatitis D
Liver disease history	07031	Viral hepatitis B without hepatic coma acute or unspecified with hepatitis D
Liver disease history	07032	Chronic viral hepatitis B without hepatic coma without hepatitis delta
Liver disease history	07033	Chronic viral hepatitis B without hepatic coma with hepatitis delta
Liver disease history	0705	Other specified viral hepatitis without mention of hepatic coma
Liver disease history	07051	Acute hepatitis C without mention of hepatic coma
Liver disease history	07052	Hepatitis D without active hepatitis B disease or hepatic coma
Liver disease history	07053	Hepatitis E without hepatic coma
Liver disease history	07054	Chronic hepatitis C without hepatic coma
Liver disease history	07059	Other specified viral hepatitis without hepatic coma
Liver disease history	0707	Unspecified viral hepatitis C
Liver disease history	07070	Unspecified viral hepatitis C without hepatic coma
Liver disease history	0709	Unspecified viral hepatitis without hepatic coma
Liver disease history	07271	Mumps hepatitis
Liver disease history	09162	Secondary syphilitic hepatitis
Liver disease history	0953	Syphilis of liver
Liver disease history	1220	Echinococcus granulosus infection of liver
Liver disease history	1225	Echinococcus multilocularis infection of liver
Liver disease history	1228	Echinococcosis unspecified of liver
Liver disease history	1305	Hepatitis due to toxoplasmosis
Liver disease history	2115	Benign neoplasm of liver and biliary passages
Liver disease history	2353	Neoplasm of uncertain behavior of liver and biliary passages
Liver disease history	571	Chronic liver disease and cirrhosis
Liver disease history	5710	Alcoholic fatty liver
Liver disease history	5711	Acute alcoholic hepatitis
Liver disease history	5712	Alcoholic cirrhosis of liver
Liver disease history	5713	Alcoholic liver damage unspecified
Liver disease history	5714	Chronic hepatitis
Liver disease history	57140	Chronic hepatitis unspecified
Liver disease history	57141	Chronic persistent hepatitis
Liver disease history	57142	Autoimmune hepatitis
Liver disease history	57149	Other chronic hepatitis
Liver disease history	5715	Cirrhosis of liver without alcohol
Liver disease history	5716	Biliary cirrhosis
Liver disease history	5718	Other chronic nonalcoholic liver disease
Liver disease history	5719	Unspecified chronic liver disease without alcohol
Liver disease history	572	Liver abscess and sequelae of chronic liver disease
Liver disease history	5720	Abscess of liver
Liver disease history	5721	Portal pyemia

Liver disease history	5728	Other sequelae of chronic liver disease
Liver disease history	573	Other disorders of liver
Liver disease history	5730	Chronic passive congestion of liver
Liver disease history	5731	Hepatitis in viral diseases classified elsewhere
Liver disease history	5732	Hepatitis in other infectious diseases classified elsewhere
Liver disease history	5733	Hepatitis unspecified
Liver disease history	5734	Hepatic infarction
Liver disease history	5738	Other specified disorders of liver
Liver disease history	5739	Unspecified disorder of liver
Liver disease history	570	Acute and subacute necrosis of liver
Liver disease history	4560	Esophageal varices with bleeding
Liver disease history	4561	Esophageal varices without bleeding
Liver disease history	4562	Esophageal varices in diseases classified elsewhere
Liver disease history	5722	Hepatic encephalopathy
Liver disease history	5723	Portal hypertension
Liver disease history	5724	Hepatorenal syndrome
Liver disease history	07041	Acute hepatitis c with hepatic coma
Liver disease history	07044	Chronic hepatitis c with hepatic coma
Liver disease history	07071	Unspecified viral hepatitis c with hepatic coma
Liver disease history	7895	Ascites
Liver disease history	45620	Esophageal varices in diseases classified elsewhere with bleeding
Liver disease history	45621	Esophageal varices in diseases classified elsewhere without bleeding
Liver disease history	78959	Other ascites
Liver disease history	V427	Liver replaced by transplant
CHF history	39891	Rheumatic heart failure (congestive)
CHF history	40201	Malignant hypertensive heart disease with heart failure
CHF history	40211	Benign hypertensive heart disease with heart failure
CHF history	40291	Unspecified hypertensive heart disease with heart failure
CHF history	40401	Hypertensive heart and chronic kidney disease malignant with heart failure with chronic kidney disease stage I through stage IV or unspecified
CHF history	40403	Hypertensive heart and chronic kidney disease malignant with heart failure with chronic kidney disease stage V or end stage renal disease
CHF history	40411	Hypertensive heart and chronic kidney disease benign with heart failure with chronic kidney disease stage I through stage IV or unspecified
CHF history	40413	Hypertensive heart and chronic kidney disease benign with heart failure with chronic kidney disease stage V or end stage renal disease
CHF history	40491	Hypertensive heart and chronic kidney disease unspecified with heart failure with chronic kidney disease stage I through stage IV or unspecified
CHF history	40493	Hypertensive heart and chronic kidney disease unspecified with heart failure with chronic kidney disease stage V or end stage renal disease
CHF history	428	Heart failure
CHF history	4280	Congestive heart failure unspecified
CHF history	4281	Left heart failure
CHF history	4282	Systolic heart failure

CHF history	4282<br>0	Unspecified systolic heart failure
CHF history	4282<br>1	Acute systolic heart failure
CHF history	4282<br>2	Chronic systolic heart failure
CHF history	4282<br>3	Acute on chronic systolic heart failure
CHF history	4283	Diastolic heart failure
CHF history	4283<br>0	Unspecified diastolic heart failure
CHF history	4283<br>1	Acute diastolic heart failure
CHF history	4283<br>2	Chronic diastolic heart failure
CHF history	4283<br>3	Acute on chronic diastolic heart failure
CHF history	4284	Combined systolic and diastolic heart failure
CHF history	4284<br>0	Unspecified combined systolic and diastolic heart failure
CHF history	4284<br>1	Acute combined systolic and diastolic heart failure
CHF history	4284<br>2	Chronic combined systolic and diastolic heart failure
CHF history	4284<br>3	Acute on chronic combined systolic and diastolic heart failure
CHF history	4289	Heart failure unspecified
CHF history	7855<br>1	Cardiogenic shock
Cerebrovascular disease history	430	Subarachnoid hemorrhage
Cerebrovascular disease history	431	Intracerebral hemorrhage
Cerebrovascular disease history	432	Other and unspecified intracranial hemorrhage
Cerebrovascular disease history	4320	Nontraumatic extradural hemorrhage
Cerebrovascular disease history	4321	Subdural hemorrhage
Cerebrovascular disease history	4329	Unspecified intracranial hemorrhage
Cerebrovascular disease history	433	Occlusion and stenosis of precerebral arteries
Cerebrovascular disease history	4330	Occlusion and stenosis of basilar artery
Cerebrovascular disease history	4330<br>0	Occlusion and stenosis of basilar artery without cerebral infarction
Cerebrovascular disease history	4330<br>1	Occlusion and stenosis of basilar artery with cerebral infarction
Cerebrovascular disease history	4331	Occlusion and stenosis of carotid artery
Cerebrovascular disease history	4331<br>0	Occlusion and stenosis of carotid artery without cerebral infarction
Cerebrovascular disease history	4331<br>1	Occlusion and stenosis of carotid artery with cerebral infarction
Cerebrovascular disease history	4332	Occlusion and stenosis of vertebral artery

Cerebrovascular disease history	43320	Occlusion and stenosis of vertebral artery without cerebral infarction
Cerebrovascular disease history	43321	Occlusion and stenosis of vertebral artery with cerebral infarction
Cerebrovascular disease history	4333	Occlusion and stenosis of multiple and bilateral precerebral arteries
Cerebrovascular disease history	43330	Occlusion and stenosis of multiple and bilateral precerebral arteries without cerebral infarction
Cerebrovascular disease history	43331	Occlusion and stenosis of multiple and bilateral precerebral arteries with cerebral infarction
Cerebrovascular disease history	4338	Occlusion and stenosis of other specified precerebral artery
Cerebrovascular disease history	43380	Occlusion and stenosis of other specified precerebral artery without cerebral infarction
Cerebrovascular disease history	43381	Occlusion and stenosis of other specified precerebral artery with cerebral infarction
Cerebrovascular disease history	4339	Occlusion and stenosis of unspecified precerebral artery
Cerebrovascular disease history	43390	Occlusion and stenosis of unspecified precerebral artery without cerebral infarction
Cerebrovascular disease history	43391	Occlusion and stenosis of unspecified precerebral artery with cerebral infarction
Cerebrovascular disease history	434	Occlusion of cerebral arteries
Cerebrovascular disease history	4340	Cerebral thrombosis
Cerebrovascular disease history	43400	Cerebral thrombosis without cerebral infarction
Cerebrovascular disease history	43401	Cerebral thrombosis with cerebral infarction
Cerebrovascular disease history	4341	Cerebral embolism
Cerebrovascular disease history	43410	Cerebral embolism without cerebral infarction
Cerebrovascular disease history	43411	Cerebral embolism with cerebral infarction
Cerebrovascular disease history	4349	Cerebral artery occlusion unspecified
Cerebrovascular disease history	43490	Cerebral artery occlusion unspecified without cerebral infarction
Cerebrovascular disease history	43491	Cerebral artery occlusion unspecified with cerebral infarction
Cerebrovascular disease history	435	Transient cerebral ischemia
Cerebrovascular disease history	4350	Basilar artery syndrome
Cerebrovascular disease history	4351	Vertebral artery syndrome
Cerebrovascular disease history	4352	Subclavian steal syndrome
Cerebrovascular disease history	4353	Vertebrobasilar artery syndrome
Cerebrovascular disease history	4358	Other specified transient cerebral ischemias
Cerebrovascular disease history	4359	Unspecified transient cerebral ischemia

Cerebrovascular disease history	436	Acute but ill-defined cerebrovascular disease
Cerebrovascular disease history	437	Other and ill-defined cerebrovascular disease
Cerebrovascular disease history	4370	Cerebral atherosclerosis
Cerebrovascular disease history	4371	Other generalized ischemic cerebrovascular disease
Cerebrovascular disease history	4373	Cerebral aneurysm nonruptured
Cerebrovascular disease history	4374	Cerebral arteritis
Cerebrovascular disease history	4375	Moyamoya disease
Cerebrovascular disease history	4376	Nonpyogenic thrombosis of intracranial venous sinus
Cerebrovascular disease history	4378	Other ill-defined cerebrovascular disease
Cerebrovascular disease history	4379	Unspecified cerebrovascular disease
Cerebrovascular disease history	438	Late effects of cerebrovascular disease
Cerebrovascular disease history	4380	Cognitive deficits
Cerebrovascular disease history	4381	Speech and language deficits
Cerebrovascular disease history	43810	Speech and language deficit unspecified
Cerebrovascular disease history	43811	Aphasia
Cerebrovascular disease history	43812	Dysphasia
Cerebrovascular disease history	43813	Dysarthria
Cerebrovascular disease history	43814	Fluency disorder
Cerebrovascular disease history	43819	Other speech and language deficits
Cerebrovascular disease history	4382	Hemiplegia/hemiparesis
Cerebrovascular disease history	43820	Hemiplegia affecting unspecified side
Cerebrovascular disease history	43821	Hemiplegia affecting dominant side
Cerebrovascular disease history	43822	Hemiplegia affecting nondominant side
Cerebrovascular disease history	4383	Monoplegia of upper limb
Cerebrovascular disease history	43830	Monoplegia of upper limb affecting unspecified side
Cerebrovascular disease history	43831	Monoplegia of upper limb affecting dominant side
Cerebrovascular disease history	43832	Monoplegia of upper limb affecting nondominant side
Cerebrovascular disease history	4384	Monoplegia of lower limb

Cerebrovascular disease history	4384 0	Monoplegia of lower limb affecting unspecified side
Cerebrovascular disease history	4384 1	Monoplegia of lower limb affecting dominant side
Cerebrovascular disease history	4384 2	Monoplegia of lower limb affecting nondominant side
Cerebrovascular disease history	4385	Other paralytic syndrome
Cerebrovascular disease history	4385 0	Other paralytic syndrome affecting unspecified side
Cerebrovascular disease history	4385 1	Other paralytic syndrome affecting dominant side
Cerebrovascular disease history	4385 2	Other paralytic syndrome affecting nondominant side
Cerebrovascular disease history	4385 3	Other paralytic syndrome bilateral
Cerebrovascular disease history	4386	Alterations of sensations
Cerebrovascular disease history	4387	Disturbances of vision
Cerebrovascular disease history	4388	Other late effects of cerebrovascular disease
Cerebrovascular disease history	4388 1	Apraxia cerebrovascular disease
Cerebrovascular disease history	4388 2	Dysphagia cerebrovascular disease
Cerebrovascular disease history	4388 3	Facial weakness
Cerebrovascular disease history	4388 4	Ataxia
Cerebrovascular disease history	4388 5	Vertigo
Cerebrovascular disease history	4388 9	Other late effects of cerebrovascular disease
Cerebrovascular disease history	4389	Unspecified late effects of cerebrovascular disease
Renal disease history	2714	Renal glycosuria
Renal disease history	2852 1	Anemia in chronic kidney disease
Renal disease history	4030 1	Hypertensive chronic kidney disease malignant with chronic kidney disease stage V or end stage renal disease
Renal disease history	4031 1	Hypertensive kidney disease benign with chronic kidney disease stage V or end stage renal disease
Renal disease history	4039 1	Hypertensive chronic kidney disease unspecified with chronic kidney disease stage V or end stage renal disease
Renal disease history	4040 2	Hypertensive heart and chronic kidney disease malignant without heart failure with chronic kidney disease stage V or end stage renal disease
Renal disease history	4040 3	Hypertensive heart and chronic kidney disease malignant with heart failure with chronic kidney disease stage V or end stage renal disease
Renal disease history	4041 2	Hypertensive heart and chronic kidney disease benign without heart failure with chronic kidney disease stage V or end stage renal disease
Renal disease history	4041 3	Hypertensive heart and chronic kidney disease benign with heart failure with chronic kidney disease stage V or end stage renal disease
Renal disease history	4049 2	Hypertensive heart and chronic kidney disease unspecified without heart failure with chronic kidney disease stage V or end stage renal disease
Renal disease history	4049 3	Hypertensive heart and chronic kidney disease unspecified with heart failure with chronic kidney disease stage V or end stage renal disease

Renal disease history	4582<br>1	Hypotension of hemodialysis
Renal disease history	580	Acute glomerulonephritis
Renal disease history	5800	Acute glomerulonephritis with lesion of proliferative glomerulonephritis
Renal disease history	5804	Acute glomerulonephritis with lesion of rapidly progressive glomerulonephritis
Renal disease history	5808	Acute glomerulonephritis with other specified pathological lesion in kidney
Renal disease history	5808<br>1	Acute glomerulonephritis in diseases classified elsewhere
Renal disease history	5808<br>9	Acute glomerulonephritis with other specified pathological lesion in kidney
Renal disease history	5809	Acute glomerulonephritis with unspecified pathological lesion in kidney
Renal disease history	581	Nephrotic syndrome
Renal disease history	5810	Nephrotic syndrome with lesion of proliferative glomerulonephritis
Renal disease history	5811	Nephrotic syndrome with lesion of membranous glomerulonephritis
Renal disease history	5812	Nephrotic syndrome with lesion of membranoproliferative glomerulonephritis
Renal disease history	5813	Nephrotic syndrome with lesion of minimal change glomerulonephritis
Renal disease history	5818	Nephrotic syndrome with other specified pathological lesion in kidney
Renal disease history	5818<br>1	Nephrotic syndrome in diseases classified elsewhere
Renal disease history	5818<br>9	Other nephrotic syndrome with specified pathological lesion in kidney
Renal disease history	5819	Nephrotic syndrome with unspecified pathological lesion in kidney
Renal disease history	582	Chronic glomerulonephritis
Renal disease history	5820	Chronic glomerulonephritis with lesion of proliferative glomerulonephritis
Renal disease history	5821	Chronic glomerulonephritis with lesion of membranous glomerulonephritis
Renal disease history	5822	Chronic glomerulonephritis with lesion of membranoproliferative glomerulonephritis
Renal disease history	5824	Chronic glomerulonephritis with lesion of rapidly progressive glomerulonephritis
Renal disease history	5828	Chronic glomerulonephritis with other specified pathological lesion in kidney
Renal disease history	5828<br>1	Chronic glomerulonephritis in diseases classified elsewhere
Renal disease history	5828<br>9	Other chronic glomerulonephritis with specified pathological lesion in kidney
Renal disease history	5829	Chronic glomerulonephritis with unspecified pathological lesion in kidney
Renal disease history	583	Nephritis and nephropathy not specified as acute or chronic
Renal disease history	5830	Nephritis and nephropathy not specified as acute or chronic with lesion of proliferative glomerulonephritis
Renal disease history	5831	Nephritis and nephropathy not specified as acute or chronic with lesion of membranous glomerulonephritis
Renal disease history	5832	Nephritis and nephropathy not specified as acute or chronic with lesion of membranoproliferative glomerulonephritis
Renal disease history	5834	Nephritis and nephropathy not specified as acute or chronic with lesion of rapidly progressive glomerulonephritis
Renal disease history	5836	Nephritis and nephropathy not specified as acute or chronic with lesion of renal cortical necrosis
Renal disease history	5837	Nephritis and nephropathy not specified as acute or chronic with lesion of renal medullary necrosis
Renal disease history	5838	Nephritis and nephropathy not specified as acute or chronic with other specified pathological lesion in kidney

Renal disease history	5838<br>1	Nephritis and nephropathy not specified as acute or chronic in diseases classified elsewhere
Renal disease history	5838<br>9	Other nephritis and nephropathy not specified as acute or chronic with specified pathological lesion in kidney
Renal disease history	5839	Nephritis and nephropathy not specified as acute or chronic with unspecified pathological lesion in kidney
Renal disease history	584	Acute kidney failure
Renal disease history	5845	Acute kidney failure with lesion of tubular necrosis
Renal disease history	5846	Acute kidney failure with lesion of renal cortical necrosis
Renal disease history	5847	Acute kidney failure with lesion of renal medullary (papillary) necrosis
Renal disease history	5848	Acute kidney failure with other specified pathological lesion in kidney
Renal disease history	5849	Acute kidney failure unspecified
Renal disease history	585	Chronic kidney disease
Renal disease history	5851	Chronic kidney disease stage I
Renal disease history	5852	Chronic kidney disease stage II (mild)
Renal disease history	5853	Chronic kidney disease stage III (moderate)
Renal disease history	5854	Chronic kidney disease stage IV (severe)
Renal disease history	5855	Chronic kidney disease stage V
Renal disease history	5856	End stage renal disease
Renal disease history	5859	Chronic kidney disease unspecified
Renal disease history	586	Renal failure unspecified
Renal disease history	587	Renal sclerosis unspecified
Renal disease history	588	Disorders resulting from impaired renal function
Renal disease history	5880	Renal osteodystrophy
Renal disease history	5881	Nephrogenic diabetes insipidus
Renal disease history	5888	Other specified disorders resulting from impaired renal function
Renal disease history	5888	Other specified disorders resulting from impaired renal function
Renal disease history	5888<br>1	Secondary hyperparathyroidism (of renal origin)
Renal disease history	5888<br>9	Other specified disorders resulting from impaired renal function
Renal disease history	5888<br>9	Other specified disorders resulting from impaired renal function
Renal disease history	5889	Unspecified disorder resulting from impaired renal function
Renal disease history	589	Small kidney of unknown cause
Renal disease history	5890	Unilateral small kidney
Renal disease history	5891	Bilateral small kidneys
Renal disease history	5899	Small kidney unspecified
Renal disease history	590	Infections of kidney
Renal disease history	5900	Chronic pyelonephritis
Renal disease history	5900<br>0	Chronic pyelonephritis without lesion of renal medullary necrosis
Renal disease history	5900<br>1	Chronic pyelonephritis with lesion of renal medullary necrosis
Renal disease history	5901	Acute pyelonephritis
Renal disease history	5901<br>0	Acute pyelonephritis without lesion of renal medullary necrosis
Renal disease history	5901<br>1	Acute pyelonephritis with lesion of renal medullary necrosis
Renal disease history	5902	Renal and perinephric abscess
Renal disease history	5903	Pyeloureteritis cystica
Renal disease history	5908	Other pyelonephritis or pyonephrosis not specified as acute or chronic
Renal disease history	5908<br>0	Pyelonephritis unspecified
Renal disease history	5908<br>1	Pyelitis or pyelonephritis in diseases classified elsewhere

Renal disease history	5909	Infection of kidney unspecified
Renal disease history	591	Hydronephrosis
Renal disease history	5920	Calculus of kidney
Renal disease history	5938<br>1	Vascular disorders of kidney
Renal disease history	5939	Unspecified disorder of kidney and ureter
Renal disease history	7910	Proteinuria
Renal disease history	7925	Cloudy (hemodialysis) (peritoneal) dialysis effluent
Renal disease history	7944	Nonspecific abnormal results of function study of kidney
Renal disease history	9965<br>6	Mechanical complication due to peritoneal dialysis catheter
Renal disease history	9966<br>8	Infection and inflammatory reaction due to peritoneal dialysis catheter
Renal disease history	9967<br>3	Other complications due to renal dialysis device implant and graft
Renal disease history	9968<br>1	Complications of transplanted kidney
Renal disease history	E870<br>2	Accidental cut puncture perforation or hemorrhage during kidney dialysis or other perfusion
Renal disease history	E871<br>2	Foreign object left in body during kidney dialysis or other perfusion
Renal disease history	E872<br>2	Failure of sterile precautions during kidney dialysis and other perfusion
Renal disease history	E874<br>2	Mechanical failure of instrument or apparatus during kidney dialysis and other perfusion
Renal disease history	E879<br>1	Kidney dialysis as the cause of abnormal reaction of patient or of later complication without misadventure at time of procedure
Renal disease history	V420	Kidney replaced by transplant
Renal disease history	V451	Renal dialysis status
Renal disease history	V451<br>1	Renal dialysis status
Renal disease history	V451<br>2	Noncompliance with renal dialysis
Renal disease history	V56	Encounter for dialysis and dialysis catheter care
Renal disease history	V560	Aftercare involving extracorporeal dialysis
Renal disease history	V561	Fitting and adjustment of extracorporeal dialysis catheter
Renal disease history	V562	Fitting and adjustment of peritoneal dialysis catheter
Renal disease history	V563	Encounter for adequacy testing for dialysis
Renal disease history	V563<br>1	Encounter for adequacy testing for hemodialysis
Renal disease history	V563<br>2	Encounter for adequacy testing for peritoneal dialysis
Renal disease history	V568	Aftercare involving other dialysis
Altered mental status	7809<br>7	Altered mental status
Respiratory rate > 29	7860<br>6	Tachypnea
SBP < 90 mm Hg	458	Hypotension
SBP < 90 mm Hg	4580	Orthostatic hypotension
SBP < 90 mm Hg	4582	Iatrogenic hypotension
SBP < 90 mm Hg	4582<br>9	Other iatrogenic hypotension
SBP < 90 mm Hg	4588	Other specified hypotension
SBP < 90 mm Hg	4589	Hypotension unspecified
SBP < 90 mm Hg	4581	Chronic hypotension

Temperature < 35°C (95°F) or > 39.9°C (103.8°F)	9916	Hypothermia
Temperature < 35°C (95°F) or > 39.9°C (103.8°F)	7806<br>5	Hypothermia not associated with low environmental temperature
Temperature < 35°C (95°F) or > 39.9°C (103.8°F)	9958<br>6	Malignant hyperthermia
Pulse<br>> 124 beats/minute	7850	Tachycardia unspecified
ph < 7.35	2762	Acidosis
Bun > 29 mg/dL	7944	Nonspecific abnormal results of function study of kidney
Sodium < 130 mg/dL	2761	Hyposmolality and/or hyponatremia
Glucose > 249 mg/dL (US) or 13.9 mmol/L (SI)	7902<br>9	Other abnormal glucose
Hct < 30%	7900<br>1	Precipitous drop in Hct
Partial pressure of oxygen < 60 mm Hg	7990<br>2	Hypoxemia
Pleural effusion on X-ray	5111	Pleurisy with effusion with a bacterial cause other than tuberculosis
Pleural effusion on X-ray	5118	Other specified forms of effusion except tuberculous
Pleural effusion on X-ray	5118<br>1	Malignant pleural effusion
Pleural effusion on X-ray	5118<br>9	Other specified forms of effusion except tuberculous
Pleural effusion on X-ray	5119	Unspecified pleural effusion

Abbreviations: BUN, blood urea nitrogen; CHF, congestive heart failure; Hct, hematocrit; HGSIL, high grade squamous intraepithelial lesion; ICD-9-CM, International Classification of Diseases, 9th revision, Clinical Modification; PSI, Pneumonia Severity Index; SBP, systemic blood pressure; SI, International System of Units; US, United States.

Table S4. 30-day mortality rates by CCI category and PSI class among patients with CABP who received ceftriaxone and macrolide on Day 1 or 2 of hospitalization.

CCI score	Overall		Risk classes I and II		Risk class III		Risk class IV		Risk class V	
	n	Mortality	n	Mortality	n	Mortality	n	Mortality	n	Mortality
**0**	7,303	0.005	4,060	0.002	2,430	0.008	796	0.011	17	0.118
**1**	9,056	0.008	4,288	0.003	3,301	0.007	1,414	0.023	53	0.057
**2**	6,295	0.014	1,876	0.005	2,462	0.011	1,854	0.022	103	0.087
**3+**	10,263	0.026	1,365	0.004	2,907	0.014	5,285	0.030	706	0.089
**Total**	32,917	0.014	11,589	0.003	11,100	0.010	9,349	0.026	879	0.087

CAP, community-acquired bacterial pneumonia; CCI, Charlson Comorbidity Index; PSI, Pneumonia Severity Index.

Table S5. 30-day CABP-related readmission rates by CCI category and PSI class among patients with CABP who received ceftriaxone and macrolide on Day 1 or 2 of hospitalization.

CCI score	Overall		Risk classes I and II		Risk class III		Risk class IV		Risk class V	
	**n**	**Readmission**	**n**	**Readmission**	**n**	**Readmission**	**n**	**Readmission**	**n**	**Readmission**
**0**	7,303	0.047	4,060	0.038	2,430	0.058	796	0.054	17	0.235
**1**	9,056	0.045	4,288	0.032	3,301	0.058	1,414	0.054	53	0.019
**2**	6,295	0.075	1,876	0.063	2,462	0.080	1,854	0.077	103	0.136
**3+**	10,263	0.102	1,365	0.095	2,907	0.090	5,285	0.108	706	0.116
**Total**	32,917	0.069	11,589	0.047	11,100	0.071	9,349	0.089	879	0.115

CAP, community-acquired bacterial pneumonia; CCI, Charlson Comorbidity Index; PSI, Pneumonia Severity Index.
